# Targeting IGF1R Overcomes Armored and Cold Tumor Microenvironment and Boosts Immune Checkpoint Blockade in Triple‐Negative Breast Cancer

**DOI:** 10.1002/advs.202501341

**Published:** 2025-07-18

**Authors:** Mengyun Wan, Jie Mei, Yun Cai, Ji Zhou, Ningyi Xue, Ying Jiang, Yan Zhang, Jingjing Huang, Yichao Zhu

**Affiliations:** ^1^ Department of Physiology School of Basic Medical Sciences Nanjing Medical University Nanjing 211166 China; ^2^ The First Clinical Medicine College Nanjing Medical University Nanjing Jiangsu 211166 China; ^3^ Department of Central Laboratory The First People's Hospital of Jintan Jintan Affiliated Hospital of Jiangsu University Changzhou Jiangsu 213251 China; ^4^ Departments of Gynecology Wuxi Maternity and Child Health Care Hospital Affiliated Women's Hospital of Jiangnan University Wuxi Jiangsu 214023 China; ^5^ Departments of Gynecology Wuxi Maternal and Child Health Care Hospital Wuxi Medical Center Nanjing Medical University Wuxi Jiangsu 214023 China; ^6^ Department of Geriatrics The Fourth Affiliated Hospital of Nanjing Medical University Nanjing Jiangsu 211800 China; ^7^ Department of General Surgery The Affiliated Taizhou People's Hospital of Nanjing Medical University Taizhou Jiangsu 214504 China

**Keywords:** armored & cold tumors, collagen, ICB, IGF1R, TNBC, tumor microenvironment

## Abstract

In the previous study, patients with tumors based on collagen deposition and immunoreactivity are classified and identified the armored & cold subtype as the most treatment‐refractory tumor type. Triple‐negative breast cancer (TNBC) is the most lethal tumor type globally, making it critical to overcome the armored and cold tumor microenvironment (TME) for effective treatment of these patients. In this study, the transcriptomic collagen activity and immune profiles of cancer patients treated with immune checkpoint blockade (ICB) are analyzed, and found that intratumoral collagen is associated with an unfavorable immunotherapeutic response and T cell exhaustion. Additionally, collagen is shown to regulate IGF1R expression at both transcriptional and post‐translational levels via SOX4 and DDR1, respectively. It is also found that IGF1R promotes tumor cell migration and invasion, as well as T cell exhaustion, with these effects mediated through collagen. Moreover, in vivo inhibition of IGF1R reversed the armored & cold TME, thereby enhancing anti‐PD‐1 therapy. In conclusion, this study identified IGF1R as a novel therapeutic target for the immuno‐collagenic subtype, and combining IGF1R inhibition with anti‐PD‐1 therapy provides a promising foundation for a novel combination immunotherapy regimen for TNBC.

## Graphical abstract

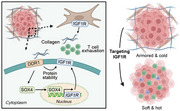

## 1 Introduction

Malignant tumors remain a global health problem with nearly 20 million new cases and 10 million cancer‐related deaths each year.^[^ ^1^ ^]^ With advancements in therapeutic strategies, immunotherapy targeting immune checkpoints has significantly transformed cancer management.^[^ ^2^, ^3^ ^]^ Although immunotherapy has ushered in a new era in cancer therapy, not all patients respond to it. Considerable efforts have been made to identify biomarkers to guide patient selection for immune checkpoint blockade (ICB), including PD‐L1 expression, which has been validated in various clinical trials.^[^ ^4^, ^5^, ^6^ ^]^ However, the limitations of PD‐L1 as the ICB biomarker have also been revealed.^[^ ^7^, ^8^, ^9^ ^]^ Increasing evidence suggests that immunotherapy response is primarily influenced by tumor microenvironment (TME) characteristics.^[^ ^10^, ^11^ ^]^ Thus, a comprehensive understanding of the TME is crucial for effective patient stratification.

Current knowledge has highlighted the two features of TME: tumor‐infiltrating immune cells (TIICs) and extracellular matrix (ECM), which are both essential for immunotherapy response.^[^ ^12^, ^13^ ^]^ Usually, TIICs abundance, represented by the cytotoxic T cell‐based immunoscore,^[^ ^14^, ^15^ ^]^ has been widely used to define hot and cold tumors, an unofficial classification of T cell inflamed and non‐inflamed tumors. In addition, the dense and rigid tumoral ECM, which is mainly structured by collagens produced by tumor cells and cancer‐associated fibroblasts (CAFs),^[^ ^16^ ^]^ contributes to patient stratification and prediction of ICB response.^[^ ^17^ ^]^ In our previous study, we developed a novel strategy to stratify patients according to collagen deposition and immune activity, and define tumors into armored & cold, soft & hot, and quiescent subtypes.^[^ ^18^ ^]^ Among these subtypes, armored & cold tumors are fatal and intractable.^[^ ^19^ ^]^ Thus, identification of novel therapeutic targets for this tumor type is of great significance to improve the prognosis of cancer patients.

Triple‐negative breast cancer (TNBC) is an aggressive subtype among all molecular types of breast cancer, which is the most prevalent tumor type worldwide.^[^ ^20^ ^]^ Due to lack of well‐established and effectively treatable targets, TNBC patients commonly exhibit the worst prognosis.^[^ ^21^, ^22^ ^]^ In the last decade, there has been a fast‐paced development in employing ICB to regulate immune responses against TNBC, in addition to conventional treatments like surgery and chemotherapy.^[^ ^23^, ^24^ ^]^ Regrettably, the positive effects of anti‐tumor immunotherapy are confined to a limited number of TNBC patients.^[^ ^24^ ^]^ Given the pan‐cancer applicability of immuno‐collagenic subtype, the identification of novel therapeutic targets modulating immunotherapy efficacy based on this subtype still needs to be further investigated.

In this study, by analyzing transcriptional associations with immuno‐collagenic features and collagen‐induced transcriptional alterations, we identified IGF1R—which is highly expressed in armored & cold tumors—as a novel therapeutic target in triple‐negative breast cancer (TNBC). IGF1R was upregulated by collagen and was found to promote tumor aggressiveness and immune evasion. Mechanistically, collagen enhanced IGF1R expression at both the transcriptional and post‐translational levels through the regulation of SOX4 and DDR1, respectively.Taken together, our findings identify IGF1R as a collagen‐induced driver of the immuno‐collagenic tumor phenotype and provide a rationale for the development of combination immunotherapeutic strategies targeting IGF1R in TNBC.

## 2 Results

### 2.1 Intratumoral Collagen was Related to Worse Therapeutic Responses and Promoted T Cells Exhaustion

**Figure 1:**
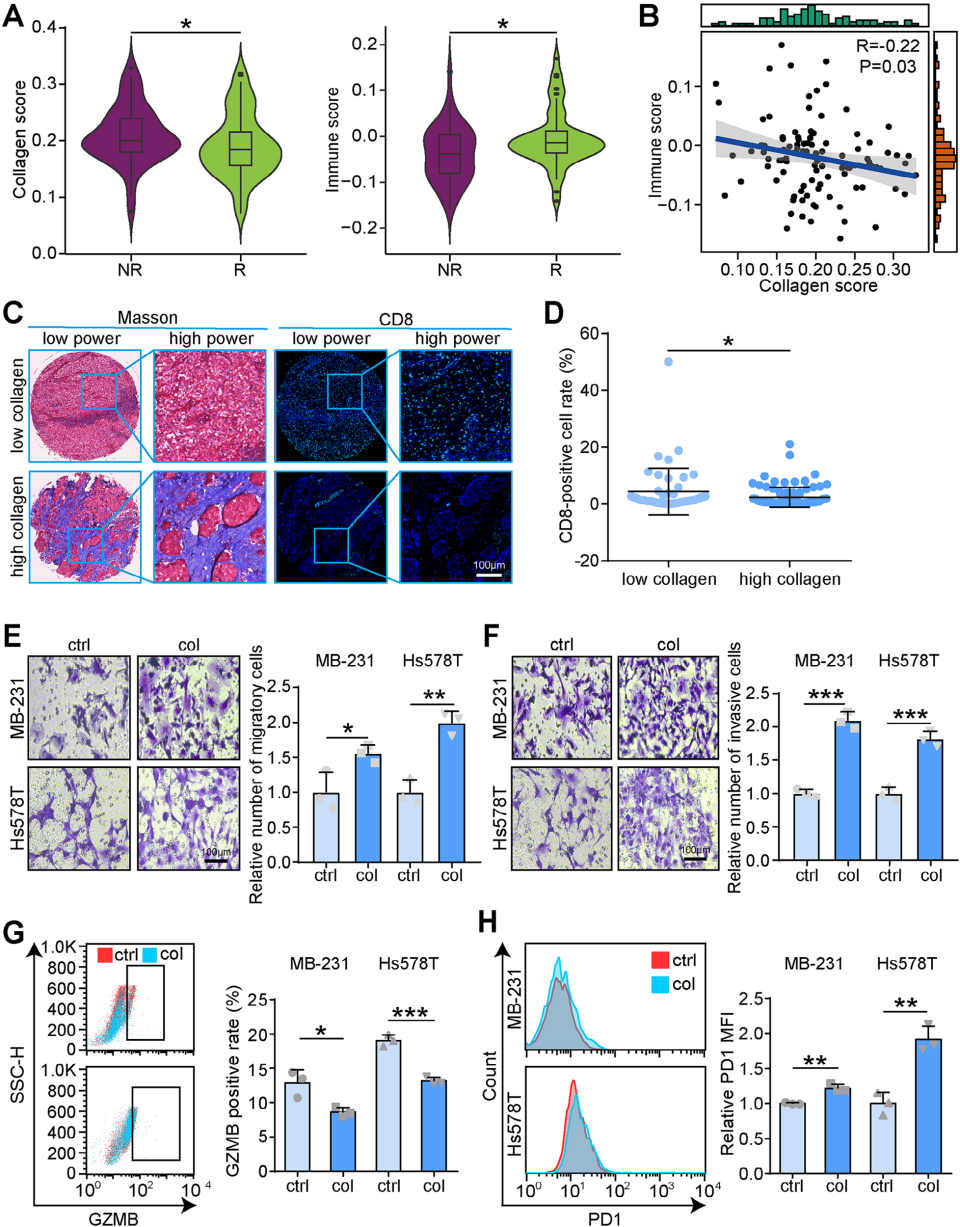
Collagen enhances tumor cells malignancy and promotes T cells exhaustion. A) Differences in collagen and immune scores in tumors from responders and non‐responders in the merged TNBC immunotherapy cohort. Significance was calculated with Student's *t*‐test. *p < 0.05. B) Correlation between collagen and immune scores in tumors from responders and non‐responders in the merged TNBC immunotherapy cohort. Significance was calculated with Pearson test. C,D) Representative images uncovering collagen and CD8 expression in the in‐house TNBC cohort and quantitative analysis. Significance was calculated with Mann‐Whitney test. *p < 0.05. E,F) The migration and invasion of MDA‐MB‐231 and Hs578T cells in control (ctrl) and collagen (col) processing groups were assessed by Boyden chamber assays. Data were presented as mean ± SD. Significance was calculated with Student's *t*‐test. All experiments were performed three times. *p < 0.05, **p < 0.01, ***p < 0.001. G,H) Flow cytometry was performed to detect GZMB and PD1 levels in the ctrl and collagen‐treated groups. Data were presented as mean ± SD. Significance was calculated with Student's *t*‐test. All experiments were performed three times. ***p < 0.001.

To investigate the role of collagen in tumor progression and immune response, we first evaluated the correlation between collagen levels and immune scores in a combined cohort of triple‐negative breast cancer (TNBC) patients undergoing immunotherapy. Tumors from non‐responders exhibited significantly higher collagen levels and lower immune scores compared to those from responders, suggesting that elevated collagen levels may be associated with poor therapeutic outcomes (**Figure** **1**A). Further correlation analysis revealed a negative relationship between collagen levels and immune scores across all samples, supporting the hypothesis that collagen‐rich tumor microenvironments are immunosuppressive (Figure 1B). To further examine the effects of collagen on immune cell infiltration, we performed immunohistochemical staining to assess collagen and CD8 expression in an in‐house TNBC cohort. Representative images showed that tumors with higher collagen content had reduced CD8+ T cell infiltration. Quantitative analysis confirmed a significant reduction in the CD8+ T cell‐positive rate in tumors with high collagen deposition in the tumor microenvironment (Figure 1C,D). To evaluate the effects of collagen on tumor cell behavior, we conducted migration and invasion assays using MDA‐MB‐231 and Hs578T cell lines. Collagen treatment significantly enhanced the migration and invasion of MDA‐MB‐231 and Hs578T cells compared to the control group, as assessed by Boyden chamber assays (Figure 1E,F). Additionally, we assessed the proliferative capacity of these cells using clone formation and CCK‐8 assays, which revealed that collagen treatment significantly increased the proliferation of MDA‐MB‐231 and Hs578T cells compared to the control group (Figure S1A,B, Supporting Information). Furthermore, we co‐cultured tumor cells with activated T cells at a 1:10 ratio for 24 h, then isolated the T cells and evaluated T cell exhaustion by measuring GZMB and PD‐1 expression via flow cytometry. The collagen‐treated group exhibited significantly higher levels of T cell exhaustion but low activated levels compared to the control group (Figure 1G,H). In addition, T cell cytokines, including IFN‐γ and TNF‐α were lowly secreted in the culture medium in the collagen‐treated group. These results suggest that collagen promotes tumor cell aggressiveness and T cell exhaustion.

### 2.2 IGF1R was a Critical Downgrade of Collagen and Correlated with Collagen Deposition

**Figure 2:**
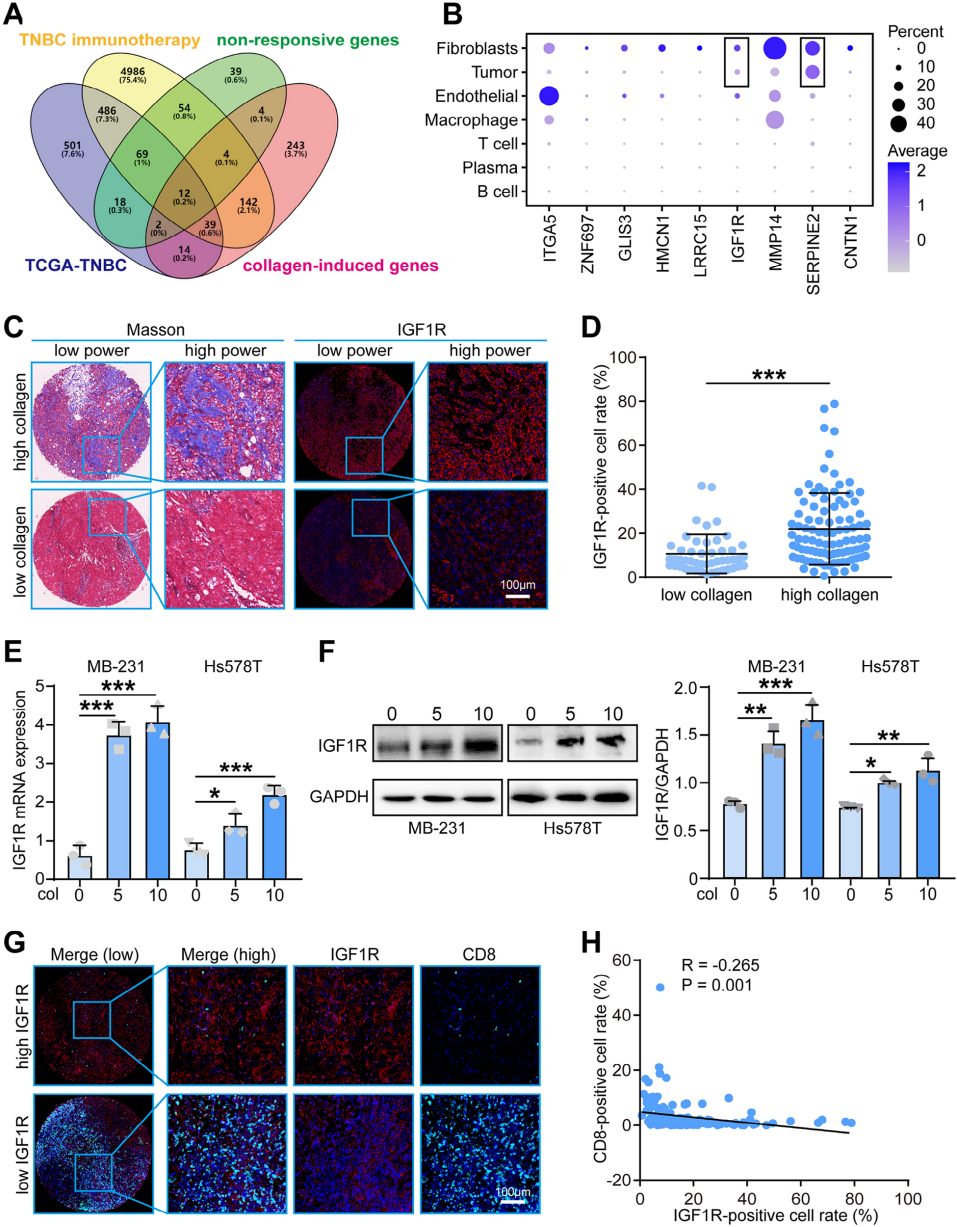
IGF1R expression and collagen content were positively correlated in TNBC. A) Venn analysis of lists of positively correlated genes with collagen score in the merged TNBC immunotherapy cohort and the TCGA‐TNBC cohort, highly expressed genes in non‐responders in the merged TNBC immunotherapy cohort, and collage‐induced genes. B) Scatter plot displaying the cell type distribution of 10 candidates in the GSE176078 dataset. C,D) Representative images uncovering collagen and IGF1R expression in the in‐house TNBC cohort and quantitative analysis. Significance was calculated with Mann‐Whitney test. ***p < 0.001. E) Evaluation of mRNA expression of IGF1R at collagen concentrations of 0, 5, and 10 µg cm^−2^ by RT‐qPCR. Data were presented as mean ± SD. Significance was calculated with One way‐ANOVA. All experiments were performed three times. *p < 0.05, ***p < 0.001. F) Protein expression of IGF1R at collagen concentrations of 0, 5, and 10 µg cm^−2^ was assessed by western blot. Data were presented as mean ± SD. Significance was calculated with One way‐ANOVA. All experiments were performed three times. *p < 0.05, **p < 0.01, ***p < 0.001. G,H) Representative images uncovering IGF1R and CD8 expression in the in‐house TNBC cohort and quantitative analysis. Significance was calculated with Pearson test.

To further investigate the relationship between collagen and immunosuppression in triple‐negative breast cancer (TNBC), we aimed to identify key genes associated with collagen expression. We performed a Venn analysis using four gene lists: genes positively correlated with collagen scores in the combined TNBC immunotherapy cohort and the TCGA‐TNBC cohort, genes highly expressed in non‐responders, and genes induced by collagen treatment. This analysis identified 12 candidate genes; however, two genes were part of the collagen score gene signature, so only 10 genes were selected for further analysis (**Figure** **2**A). Among these 10 candidate genes, IGF1R and SERPINE2 were specifically and significantly expressed in both tumor cells and fibroblasts (Figure S2A–D, Supporting Information, Figure 2B), suggesting their dual roles in the tumor and stromal compartments. However, as SERPINE2 remains in the early stages of research and lacks specific inhibitors, we selected IGF1R as the key gene for subsequent studies. To validate the correlation between collagen and IGF1R in TNBC, we examined their co‐expression in our in‐house TNBC cohort. Tumors with elevated collagen levels exhibited significantly higher IGF1R expression, reinforcing the positive correlation observed in previous analyses (Figure 2C,D). We assessed IGF1R mRNA levels by RT‐qPCR in MDA‐MB‐231 and Hs578T cells and found that IGF1R mRNA expression was significantly increased at a collagen concentration of 5 µg cm^−^^2^ compared to 0 µg cm^−^^2^, with further increases at 10 µg cm^−^^2^ (Figure 2E). Western blot analysis confirmed that IGF1R protein expression was consistent with mRNA levels, showing increased expression at 5 µg cm^−^^2^ and even higher expression at 10 µg cm^−^^2^ (Figure 2F). Finally, we investigated the functional consequences of collagen‐induced IGF1R expression on immune cell infiltration. Co‐immunofluorescence staining for IGF1R and CD8 in the in‐house TNBC cohort revealed that tumors with higher IGF1R expression had significantly reduced CD8+ T cell infiltration (Figure 2G,H). Additionally, IGF1R expression was significantly higher in tumor tissues compared to adjacent non‐tumor tissues (Figure S3, Supporting Information). These findings suggest that collagen‐induced IGF1R expression may contribute to immune suppression by limiting cytotoxic T cell infiltration within the tumor microenvironment.

### 2.3 Collagen up‐Regulated IGF1R Expression at Both Transcriptional and Post‐Translational Level

**Figure 3:**
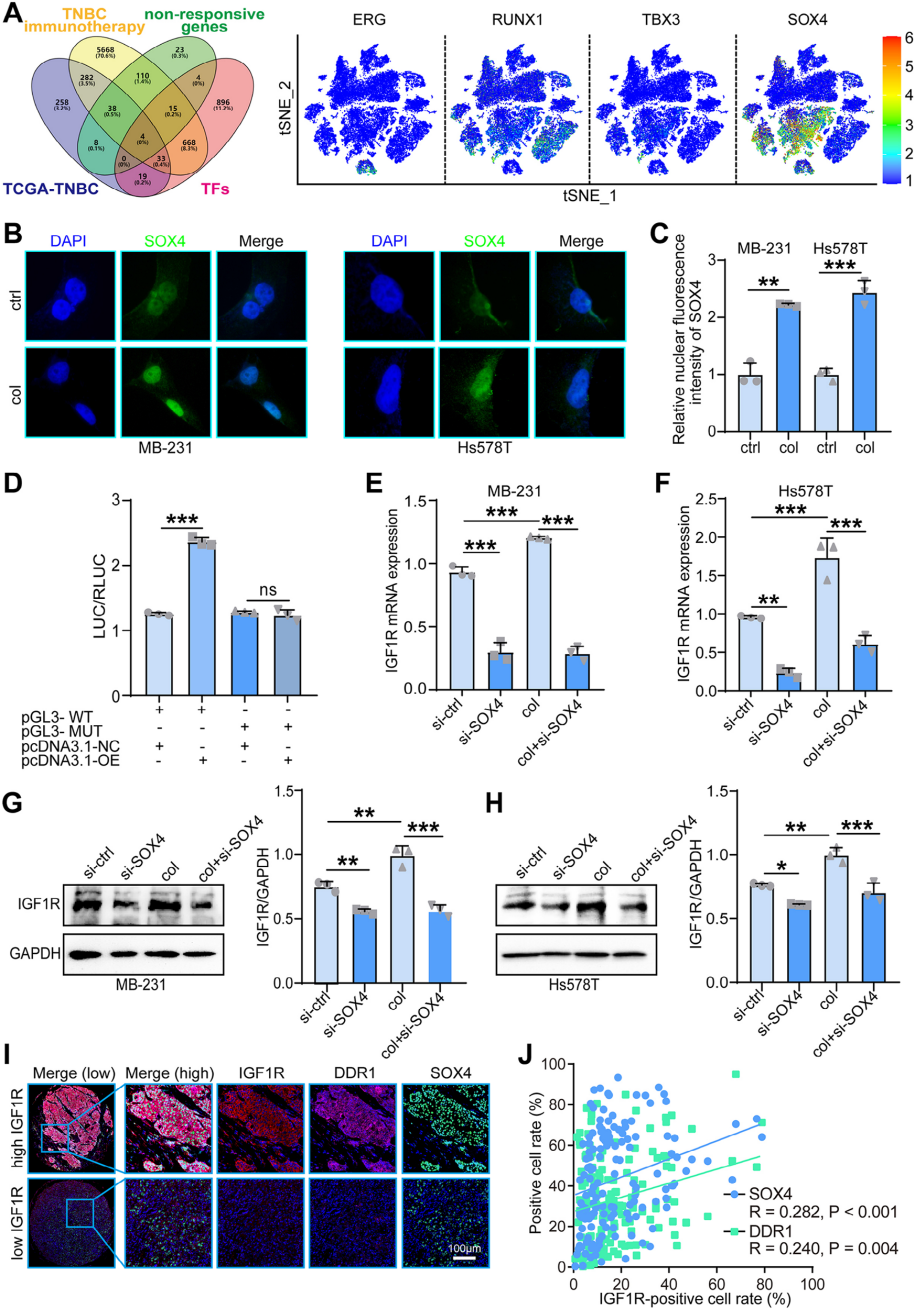
Collagen regulates the transcription factor SOX4 at the transcriptional level to regulate IGF1R expression. A) Venn analysis of lists of positively correlated genes with collagen score in the merged TNBC immunotherapy cohort and the TCGA‐TNBC cohort, highly expressed genes in non‐responders in the merged TNBC immunotherapy cohort, and transcriptional factors (left), and t‐SNE plot displaying the expression of 10 candidates in different cell types in the GSE176078 dataset (right). B,C) The location of IGF1R in control and collagen‐treated MDA‐MB‐231 and Hs578T cells was assessed by immunofluorescence staining assay. Data were presented as mean ± SD. Significance was calculated with Student's *t*‐test. All experiments were performed three times. **p < 0.01, ***p < 0.001. D) Dual‐Glo fluorokinase assay verifies direct binding of promoter IGF1R and transcription factor SOX4. Data were presented as mean ± SD. Significance was calculated with one way‐ANOVA. All experiments were performed three times. ns, non‐significance, ***p < 0.001. E,F) Evaluation of mRNA expression of IGF1R in control (si‐ctrl), knockdown SOX4 (si‐SOX4), collagen‐treated group and knockdown of SOX4 after collagen treatment groups assessed by RT‐qPCR. Data were presented as mean ± SD. Significance was calculated with one way‐ANOVA. All experiments were performed three times. **p < 0.01, ***p < 0.001. G,H) Protein expression of IGF1R in control(si‐ctrl), knockdown SOX4(si‐SOX4), collagen‐treated group and knockdown of SOX4 after collagen treatment groups assessed by western blot. Data were presented as mean ± SD. Significance was calculated with one way‐ANOVA. All experiments were performed three times. *p < 0.05, **p < 0.01, ***p < 0.001. I,J) Representative images uncovering IGF1R, DDR1, and SOX4 expression in the in‐house TNBC cohort and quantitative analysis. Significance was calculated with Pearson test.

To further investigate the transcriptional regulation of IGF1R by collagen, we conducted a Venn analysis to identify transcription factors associated with collagen content, immunotherapy non‐responsiveness, and triple‐negative breast cancer (TNBC) cohorts. Ten candidate transcription factors were identified and visualized using at‐SNE plot, revealing their cell‐type distribution in the GSE176078 dataset. Among these, SOX4 was expressed in both tumor cells and fibroblasts and was selected for further study (**Figure** **3**A). Immunofluorescence staining showed that collagen treatment increased SOX4 nuclear localization in MDA‐MB‐231 and Hs578T cells compared to the control group (Figure 3B,C), indicating that collagen promotes SOX4 nuclear translocation. A dual‐luciferase reporter assay confirmed that SOX4 could regulate IGF1R expression (Figure 3D), establishing SOX4 as a potential upstream transcription factor of IGF1R. We verified the knockdown efficiency of SOX4‐targeting siRNAs by Western blot (Figure S4, Supporting Information). RT‐qPCR analysis revealed that IGF1R mRNA levels in MDA‐MB‐231 and Hs578T cells were reduced following SOX4 knockdown compared to the control group. In contrast, collagen stimulation increased IGF1R mRNA levels, while SOX4 knockdown in collagen‐stimulated cells reduced IGF1R mRNA levels compared to the collagen group (Figure 3E,F). Western blot analysis confirmed that IGF1R protein expression was consistent with mRNA levels (Figure 3G,H). These results suggest that collagen regulates IGF1R expression at the transcriptional level through the transcription factor SOX4.

**Figure 4:**
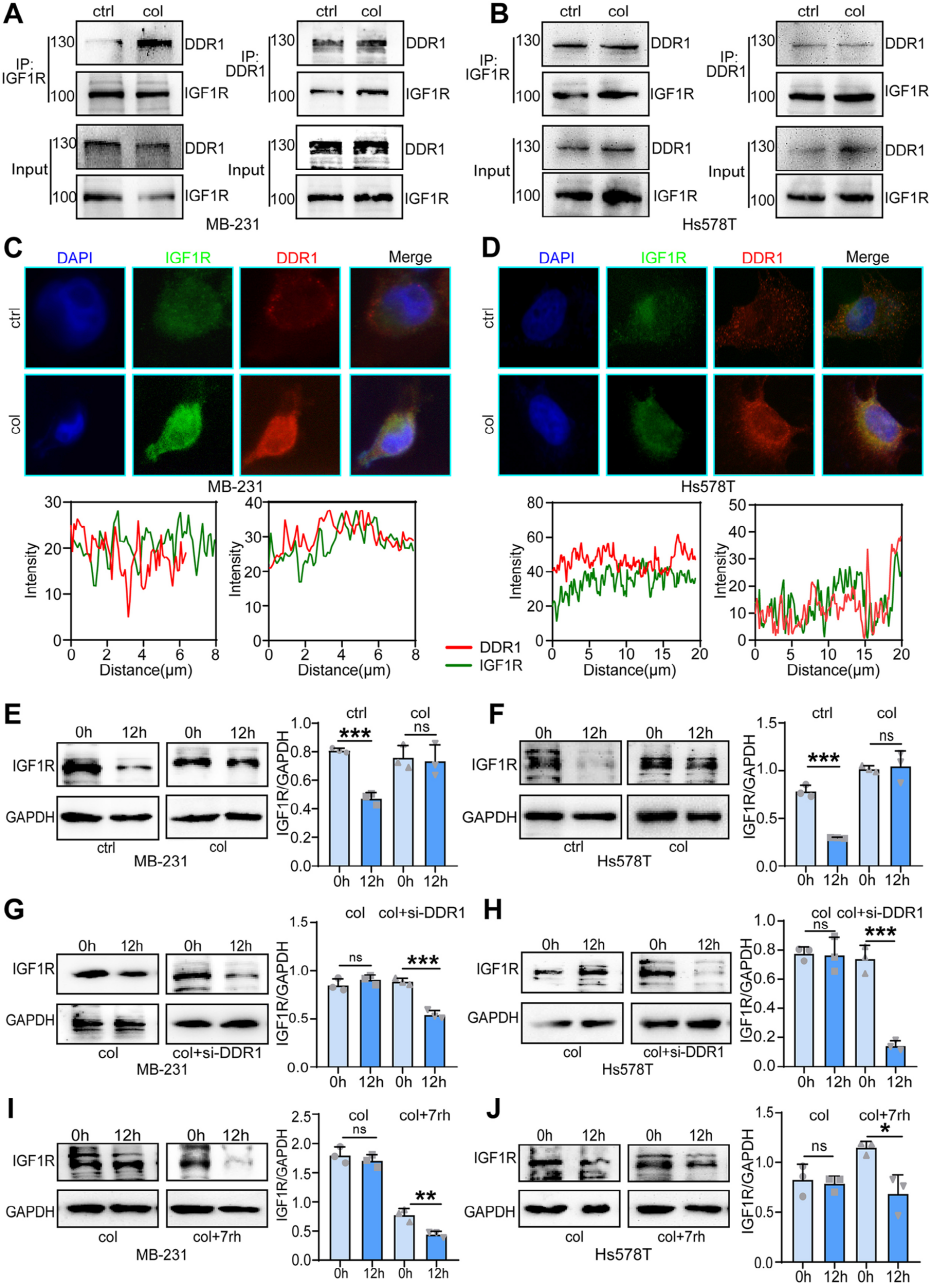
IGF1R interacts with the collagen receptor DDR1 and protects it from degradation. A,B) CO‐IP assay to verify whether IGF1R and collagen receptor DDR1 interact with each other. C,D) Immunofluorescence assay to verify the promotion of co‐localization of IGF1R and DDR1 in the cell membrane at a collagen concentration of 10µg cm^−2^. E,F) MDA‐MB‐231 and Hs578T cells were treated with ctrl and collagen. Chlordiazepoxide (CHX, 100 µg mL^−1^) was used to inhibit protein synthesis. Cellular proteins were extracted at 0 and 12 h after CHX treatment and Western blotting was performed to detect IGF1R expression. Data were presented as mean ± SD. Significance was calculated with Student's *t*‐test. All experiments were performed three times. ns, non‐significance, ***p < 0.001. G,H) MDA‐MB‐231 and Hs578T cells were treated with collagen and knockdown of DDR1 (si‐DDR1) after collagen treatment. Chlordiazepoxide (CHX, 100 µg mL^−1^) was used to inhibit protein synthesis. Cellular proteins were extracted at 0 and 12 h after CHX treatment and Western blotting was performed to detect IGF1R expression. Data were presented as mean ± SD. Significance was calculated with Student's *t*‐test. All experiments were performed three times. ns, non‐significance, ***p < 0.001. I,J) MDA‐MB‐231 and Hs578T cells were treated with collagen and 7rh and collagen co‐ treated. Chlordiazepoxide (CHX, 100 µg mL^−1^) was used to inhibit protein synthesis. Cellular proteins were extracted at 0 and 12 h after CHX treatment and Western blotting was performed to detect IGF1R expression. Data were presented as mean ± SD. Significance was calculated with Student's *t*‐test. All experiments were performed three times. ns, non‐significance, *p < 0.05, **p < 0.01.

Given that DDR1 is a known collagen receptor that mediates collagen‐induced signaling, we evaluated its expression alongside SOX4 and IGF1R. Immunohistochemical analysis of our in‐house triple‐negative breast cancer (TNBC) cohort revealed a positive correlation among IGF1R, DDR1, and SOX4 expression, which was further validated by Pearson correlation analysis in tumor tissues (Figure 3I,J). To investigate the interaction between IGF1R and DDR1, co‐immunoprecipitation (Co‐IP) assays confirmed their direct binding, and immunofluorescence staining demonstrated that collagen treatment enhanced the co‐localization of IGF1R and DDR1 at the cell membrane in MDA‐MB‐231 and Hs578T cells (**Figure** **4**A–D, Supporting Information). To assess protein stability, we inhibited protein synthesis using cycloheximide (CHX, 100 µg mL^−1^, Cat. No. HY‐12320, MedChemExpress, Shanghai, China) in MDA‐MB‐231 and Hs578T cells, extracted proteins at 0 and 12 h post‐CHX treatment, and performed Western blot analysis to detect IGF1R expression. Collagen treatment significantly slowed IGF1R degradation (Figure 4E,F). In additional experiments with CHX treatment, we examined proteins at 0 and 12 h and found that DDR1 knockdown in collagen‐treated cells reduced DDR1 expression and accelerated IGF1R degradation compared to the collagen‐only group in MDA‐MB‐231 and Hs578T cells (Figure 4G,H). These results suggest that DDR1 and IGF1R interact to stabilize each other, preventing IGF1R degradation. Beyond its role in IGF1R stabilization, DDR1 also modulates downstream signaling pathways. In MDA‐MB‐231 and Hs578T cells treated with collagen, DDR1 knockdown or treatment with the DDR1 inhibitor 7rh (Cat. No. I9783, Sigma‐Aldrich, Saint Louis, USA) reduced collagen‐mediated SOX4 nuclear localization (Figure S5A,B, Supporting Information). In addition, RT‐qPCR and Western blot analysis revealed that IGF1R levels in TNBC cells were reduced following DDR1 knockdown or DDR1 inhibition compared to the control group. In contrast, collagen stimulation increased IGF1R levels, while DDR1 knockdown or DDR1 inhibition in collagen‐stimulated cells reduced IGF1R levels compared to the collagen group (Figure S6A–H, Supporting Information). Moreover, in vivo SOX4 and DDR1 knockdown both inhibited tumor growth and IGF1R expression in tumor tissues (Figure S7A,B, Supporting Information). Additionally, SOX4 and DDR1 expression levels were significantly higher in tumor tissues compared to adjacent non‐tumor tissues (Figure S8A,B, Supporting Information). These findings indicate that SOX4 acts downstream of DDR1 and that collagen regulates SOX4 nuclear localization by modulating DDR1 function.

### 2.4 IGF1R Accelerate Tumor Cell Aggressive and Promoted T Cells Exhaustion

**Figure 5:**
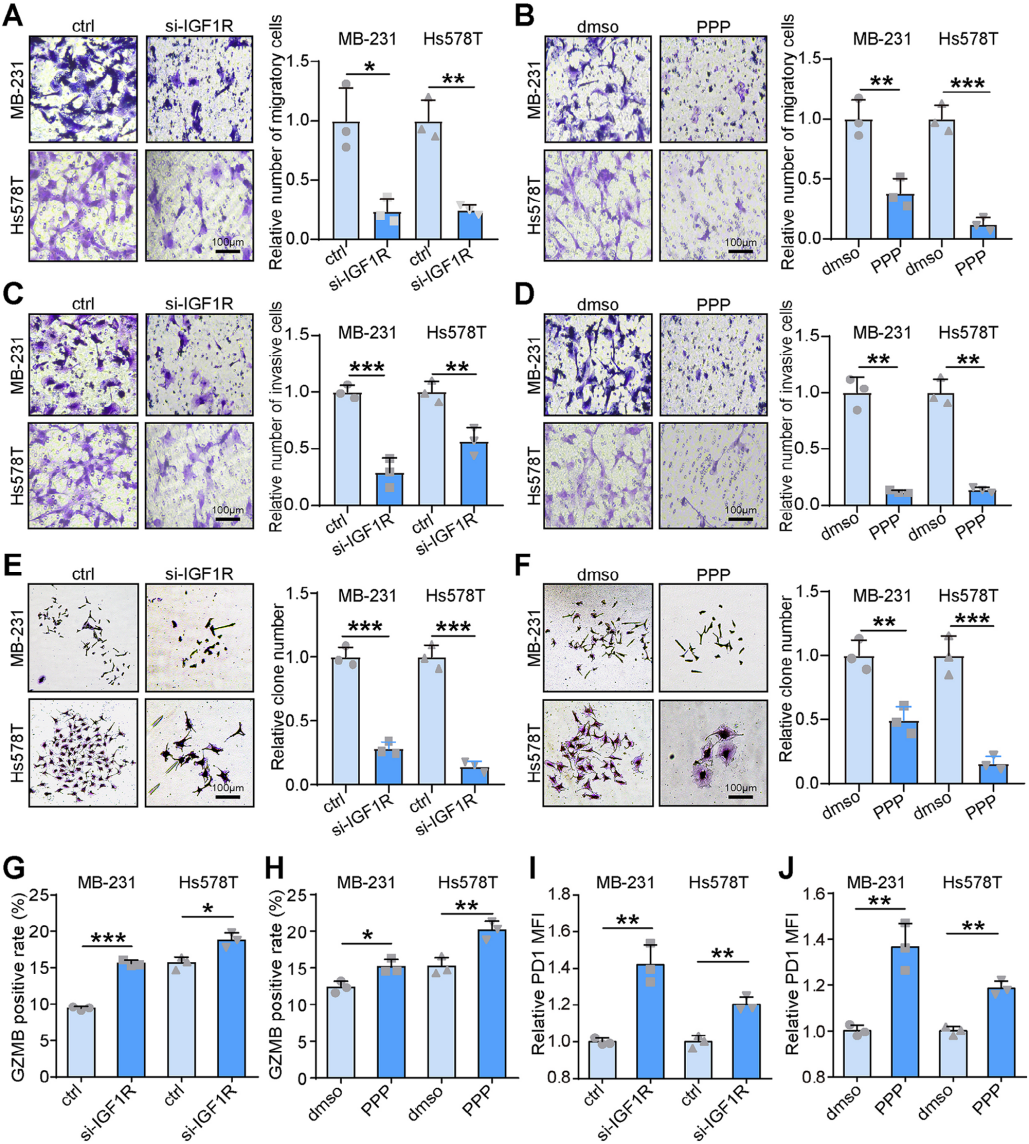
Inhibition of IGF1R expression suppresses tumor development and reduces T cell depletion. A–D) The migration and invasion of MDA‐MB‐231 and Hs578T cells in control (si‐ctrl), knockdown IGF1R (si‐IGF1R), dmso, and PPP (IGF1R inhibitor)‐treated groups were assessed by Boyden chamber assays. Data were presented as mean ± SD. Significance was calculated with Student's *t*‐test and Mann‐Whitney test. All experiments were performed three times. *p < 0.05, **p < 0.01, ***p < 0.001. E,F) The proliferation of MDA‐MB‐231 and Hs578T cells in si‐ctrl, si‐IGF1R, dmso, and PPP‐treated groups were assessed by colony formation assays. Data were presented as mean ± SD. Significance was calculated with Student's *t*‐test. All experiments were performed three times. **p < 0.01, ***p < 0.001. G–J) Flow cytometry was performed to detect GZMB and PD1 levels in the si‐ctrl, si‐IGF1R, dmso, and PPP‐treated groups. Data were presented as mean ± SD. Significance was calculated with Student's *t*‐test. All experiments were performed three times. *p < 0.05, **p < 0.01, ***p < 0.001.

We wanted to know the effect on tumor cells after directly affecting IGF1R. First we found that knockdown of IGF1R or direct treatment with Picropodophyllin (PPP), an inhibitor of IGF1R, inhibited the migration and invasion of MDA‐MB‐231 and Hs578T cells by Boyden chamber experiments (**Figure** **5**A–D). Then it was found by clone formation experiments that knockdown of IGF1R or use of PPP inhibited the proliferative ability of MDA‐MB‐231 and Hs578T (Figure 5E,F). The results of CCK8 experiments were found to be consistent with the results of plate cloning integration experiments (Figure S9A,B, Supporting Information). To investigate the impact of IGF1R on T cells, we co‐cultured T cells with MDA‐MB‐231 or Hs578T cells at a 1:10 ratio, followed by isolation of the T cells. The expression of GZMB and PD1 in the isolated T cells was assessed using flow cytometry. Our results showed that both IGF1R knockdown and PPP treatment enhanced GZMB expression but reduced PD1 expression in T cells (Figure 5G–J). In addition, both IGF1R knockdown and PPP treatment enhanced the levels of IFN‐γ and TNF‐α in the culture medium (Figure S9C,D, Supporting Information). All data indicated that IGF1R plays a role in promoting T cell exhaustion.

### 2.5 IGF1R Regulated Collagen‐Induced Tumor Cell Aggressive and Promoted T Cells Exhaustion

We found through Boyden chamber experiments that in MDA‐MB‐231 and Hs578T cells, knockdown of IGF1R after collagen treatment, or collagen treatment followed by the IGF1R inhibitor PPP, significantly attenuated cell migration and invasion compared to collagen treatment alone (Figure S10A,B, Supporting Information). The results of the CCK8 assays were consistent with those of the clone formation assays (Figure S10C,D, Supporting Information). Subsequently, clone formation assays showed that collagen treatment followed by IGF1R knockdown, or collagen treatment followed by PPP administration, also significantly reduced the proliferative capacity of the cells compared to collagen treatment alone (Figure S10E,F, Supporting Information). We then co‐cultured T cells with MDA‐MB‐231 or Hs578T cells at a ratio of 1:10, isolated the T cells, and detected GZMB and PD1 expression in the isolated T cells by flow cytometry. We found that both IGF1R knockdown after collagen treatment and PPP administration after collagen treatment increased GZMB expression but decreased PD1 expression in T cells compared to collagen treatment alone (Figure S10G–J, Supporting Information). In addition, both IGF1R knockdown after collagen treatment and PPP administration after collagen treatment increased the levels of IFN‐γ and TNF‐α in the culture medium (Figure S10K,L, Supporting Information). These results indicate that collagen mediates IGF1R signaling to promote the malignant phenotype of tumor cells and contributes to T cell exhaustion.

### 2.6 Inhibition of IGF1R Suppresses Collagen and Migration in Cancer‐Associated Fibroblasts

**Figure 6:**
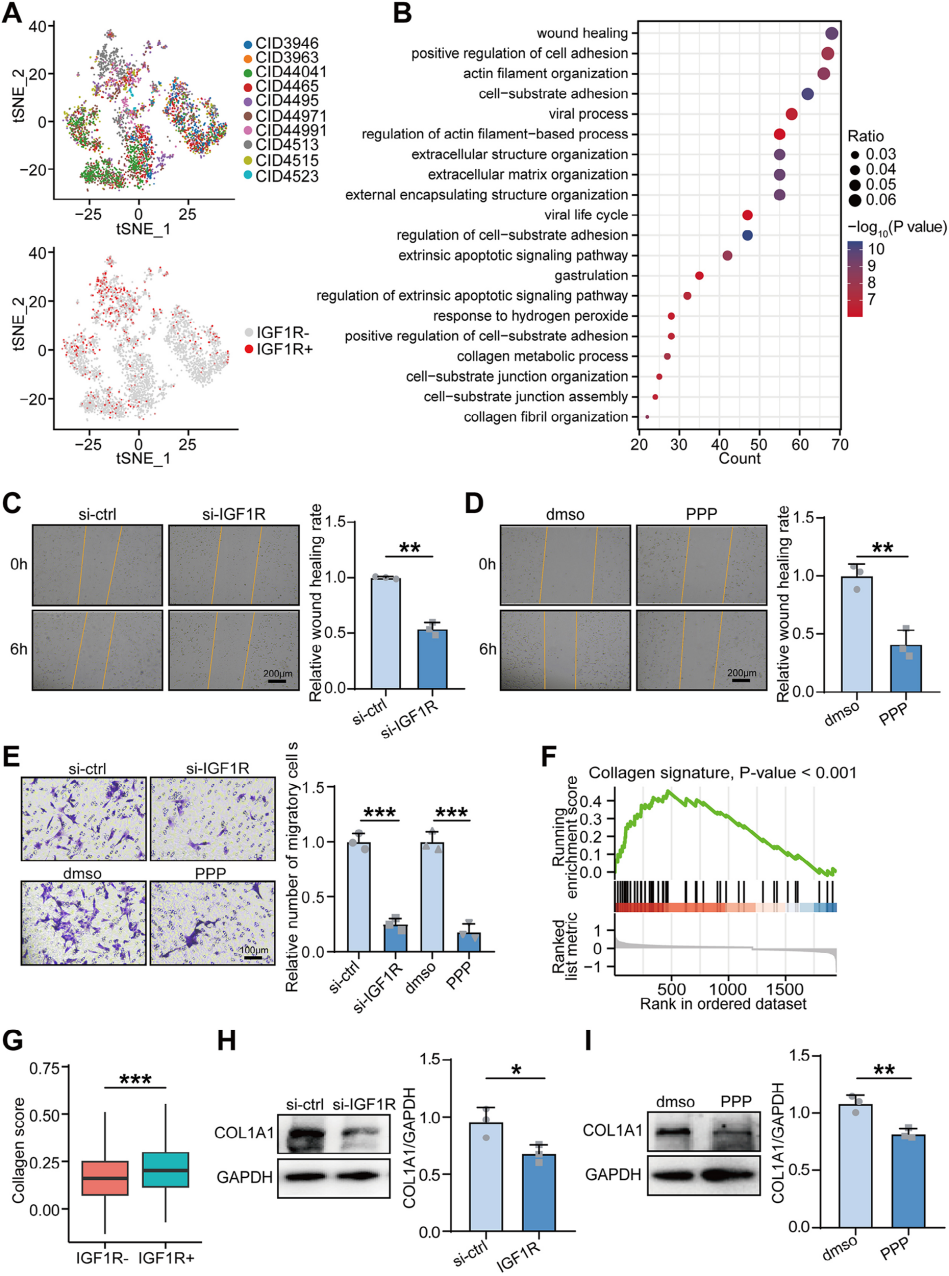
Inhibition of IGF1R expression suppresses collagen expression and migration of cancer‐associated fibroblasts (CAFs). A) t‐SNE visualization of single cells passed quality controls, colored by 10 TNBC patients in the GSE176078 cohort, and expression pattern of IGF1R in fibroblasts overlaid on t‐SNE. Red points represent the cells with AGTR1 expression. B) Functional enrichment analysis of genes highly expressed on IGF1R+ fibroblasts in the term of Gene Ontology biologocal process. C,D) The migration of fibroblasts in control (si‐ctrl), knockdown IGF1R (si‐IGF1R), dmso, and PPP (IGF1R inhibitor)‐treated groups were assessed by Wound‐healing assay. Data were presented as mean ± SD. Significance was calculated with Student's *t*‐test. All experiments were performed three times. **p < 0.01. E) The migration of fibroblasts in si‐ctrl, si‐IGF1R, dmso, and PPP‐treated groups were assessed by Boyden chamber assays. Data were presented as mean ± SD. Significance was calculated with Student's *t*‐test. All experiments were performed three times. ***p < 0.001. F) GSEA of collagen signature between fibroblasts with positive and negative IGF1R. G) Differences in collagen score in fibroblasts with positive and negative IGF1R. H,I) Protein expression of COL1A1 in si‐ctrl, si‐IGF1R, dmso, and PPP‐treated groups assessed by western blot. Data were presented as mean ± SD. Significance was calculated with Student's *t*‐test. All experiments were performed three times. *p < 0.05, **p < 0.01.

To explore the role of IGF1R in cancer‐associated fibroblasts (CAFs), t‐SNE analysis of single‐cell RNA‐seq data from TNBC patients (GSE176078) revealed high IGF1R expression in fibroblasts (**Figure** **6**A). Functional enrichment analysis showed that genes highly expressed in IGF1R⁺ fibroblasts were associated with extracellular matrix organization, cell migration, and other biological processes related to tumor progression (Figure 6B).To evaluate the functional effects of IGF1R inhibition on fibroblast migration, wound healing and Boyden chamber assays demonstrated that both IGF1R knockdown and treatment with the IGF1R inhibitor PPP significantly suppressed CAF migration (Figure 6C–E).To further investigate the effect of IGF1R on collagen production in CAFs, gene set enrichment analysis (GSEA) revealed a positive correlation between IGF1R expression and a collagen‐related gene signature in fibroblasts (Figure 6F). In addition, IGF1R⁺ fibroblasts exhibited significantly higher collagen scores (Figure 6G).Western blot analysis further confirmed that both IGF1R knockdown and PPP treatment markedly reduced the protein expression of COL1A1, a major collagen component, compared to controls (Figure 6H,I).These findings suggest that IGF1R expression in CAFs promotes both collagen production and fibroblast migration, highlighting IGF1R as a potential target for reducing the pro‐tumorigenic activity of CAFs in the tumor microenvironment.

### 2.7 In Vivo Inhibition of IGF1R Reversed the Armored & Cold TME and Boosted Anti‐PD‐1 Therapy

**Figure 7:**
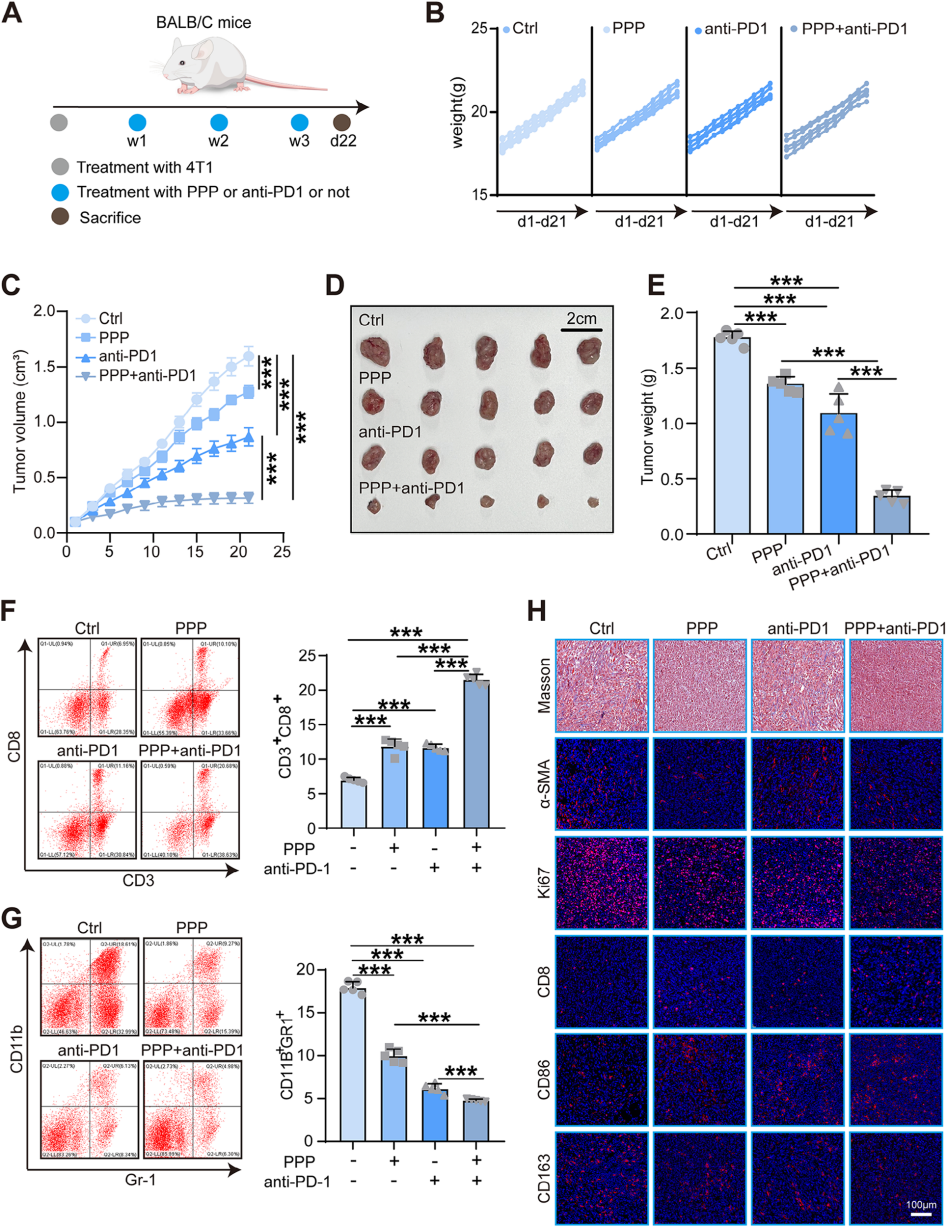
In vivo inhibition of IGF1R reversed immuno‐cold TME and boosted anti‐PD‐1 therapy. A) Schematic diagram of mouse model construction. B) Weight gain curves of mice treated with PBS, Picropodophyllin (PPP), anti‐PD1 antibody, and PPP combined with anti‐PD1 antibody for 21 days. C) Tumor volume growth curves of mice treated with PBS, Picropodophyllin (PPP), anti‐PD1 antibody, and PPP combined with anti‐PD1 antibody for 21 days. D,E) Representative images showing the tumors harvested from mice bearing 4T1 cells treated with PBS, PPP, anti‐PD‐1 antibody, and the combination, and weight of the harvested tumors. Data were presented as mean ± SD. Significance was calculated with one‐way ANOVA. ***p < 0.001. F) Representative results of flow cytometry analysis of total CD8^+^T cells represented by CD3^+^CD8^+^and quantitative analysis. Data were presented as mean ± SD. Significance was calculated with one‐way ANOVA. ***P < 0.001. G) Representative results of flow cytometry analysis of myeloid‐derivied suppressor cells (MDSC) represented by CD11b^+^Gr‐1^+^ and quantitative analysis. Significance was calculated with one‐way ANOVA. ns, non‐significance, ***p < 0.001. H) Representative images showing collagen levels and expression of α‐SMA, Ki67, CD8, CD86, and CD163 in tumor tissues from different groups of mice.

To investigate the effect of IGF1R on the tumor microenvironment (TME) in vivo, we established a mouse model (**Figure** **7**A). Specifically, 4T1 cells were subcutaneously injected into mice to generate a breast cancer model, and tumor‐bearing mice were randomly divided into four groups: control, PPP treatment, anti‐PD1 treatment, and combination treatment with PPP and anti‐PD1.Body weights of the mice were monitored from day 1 to day 21 and showed no significant differences among the four groups (Figure 7B). Tumor volumes were also measured over this period, and growth curves were plotted accordingly (Figure 7C). Tumor growth was significantly suppressed in the PPP, anti‐PD1, and combination treatment groups, with the most pronounced reduction observed in the group receiving combined PPP and anti‐PD1 therapy. Tumor weights at the endpoint were consistent with the tumor volume measurements (Figure 7D,E).Flow cytometry analysis further revealed an increase in the number of CD8⁺ T cells and a decrease in myeloid‐derived suppressor cells (MDSCs) in tumors from mice treated with PPP and/or anti‐PD1, particularly in the combination group (Figure 7F,G).Collectively, these findings suggest that IGF1R inhibition can remodel the TME into a “soft and hot” phenotype, thereby enhancing the efficacy of PD1 blockade in vivo. Additionally, immunohistochemical and Masson staining of mouse tumor tissues (Figure 7H; Figure S11A–F, Supporting Information) revealed that combined PPP and anti‐PD1 treatment increased infiltration of CD8⁺ T cells and CD86⁺ M1 macrophage, suppressed Ki67 expression in tumor cells, and inhibited collagen deposition and expression CAF marker α‐SMA and M2 macrophage marker CD163, indicating both reduced tumor proliferation and altered immune activation within the TME.

### 2.8 Extension of Regulation and Predictive Role of IGF1R in Pan‐Cancer

Taken together, these data indicate that IGF1R is associated with poor immunotherapeutic responses in TNBC. To further explore the regulatory network of IGF1R, we analyzed its correlation with two key regulators, SOX4 and DDR1. Across most cancer types, IGF1R expression was positively correlated with both SOX4 and DDR1, with the exception of a few tumor types (Figure S12A,B, Supporting Information). Additionally, we assessed the relationship between IGF1R expression and immunotherapeutic efficacy. High IGF1R expression was found to predict poor immunotherapeutic responses in the GSE176307 cohort (bladder cancer), the IMvigor210 cohort (bladder cancer), and the GSE126044 cohort (non‐small cell lung cancer) (Figure S12C–E, Supporting Information).These findings suggest that IGF1R may serve as a pan‐cancer predictive biomarker for immunotherapy response, with potential exceptions in certain cancer types.

## 3 Discussion

Our study found that higher levels of collagen in tumors lead to increased T cell exhaustion, prompting immune escape of tumor cells and thus less effective immunotherapy. Numerous high‐impact studies have established collagen's role in promoting immune evasion. For example, there are findings confirming that collagen limits T‐cell infiltration in pancreatic cancer through matrix remodeling, and there are studies linking collagen deposition to immunotherapy resistance in lung cancer.^[^ ^17^, ^25^ ^]^ These seminal works highlight collagen as a critical immunosuppressive factor, primarily through physical barriers or stromal effects. In addition, many high‐profile studies have elucidated collagen's impact on immune evasion from the perspective of immune cells. It has been shown that collagen induces T‐cell exhaustion, such as PD‐1 upregulation and reduced cytokine secretion.^[^ ^26^ ^]^ Collagen‐driven polarization of M2 macrophages in breast cancer has also been reported to amplify immunosuppression.^[^ ^27^ ^]^ These studies emphasize direct effects on immune cell function, but rarely explore tumor cells as mediators of this process.Our analysis screened that IGF1R is highly expressed in tumor cells and is closely associated with collagen deposition. Through analysis and experiments we concluded that collagen regulates IGF1R at the transcriptional level through SOX4, and that the collagen receptor DDR1 binds to IGF1R to stabilize its structure. Meanwhile, we experimentally found that knockdown of IGF1R and the use of Picropodophyllin, an inhibitor of IGF1R, suppressed the proliferation, migration, and invasion of TNBC cells, and inhibited immune escape. Finally, we found through a series of experiments that IGF1R is mediated by collagen to achieve its effect on tumor cells. In in vivo experiments, we discovered that inhibition of IGF1R reversed armored & cold TME and boosted anti‐PD‐1 therapy.

IGF1R is a transmembrane tyrosine kinase receptor that primarily activates downstream signaling pathways by binding to insulin‐like growth factors IGF‐1 and IGF‐2.^[^ ^28^ ^]^ These signaling pathways include two classical signaling pathways, PI3K/Akt and MAPK/ERK, through which IGF1R affects the proliferation and differentiation of tumor cells.^[^ ^29^, ^30^ ^]^ In a variety of tumors, high levels of IGF1R expression are closely associated with the degree of tumor malignancy, metastatic ability, and patient prognosis.^[^ ^31^, ^32^ ^]^ It has been shown that in the tumor microenvironment, IGF1R works as a cancer‐promoting factor to promote tumor cell development.^[^ ^28^ ^]^ In addition, IGF1R interacts with the ECM, fibroblasts, and other components of the tumor microenvironment to promote tumor angiogenesis.^[^ ^33^, ^34^, ^35^ ^]^ Several studies have demonstrated that IGF1R signaling influences the immune microenvironment, primarily through its effects on immune cell function and tumor‐immune crosstalk. Liu et al. revealed that macrophage promote tumor progression via IGF1‐IGF1R interaction in melanoma.^[^ ^36^ ^]^ Song et al. revealed insulin‐like growth factor 2 drives fibroblast‐mediated tumor immunoevasion and confers resistance to immunotherapy via IGF1R.^[^ ^37^ ^]^ Many recent studies have found that blocking the IGF1R signaling pathway can effectively inhibit tumor growth and metastasis, and plays a key role in preventing malignant transformation of tumors.^[^ ^32^ ^]^ Meanwhile, inhibition of IGF1R alone or in combination with chemotherapy, radiotherapy, and immunotherapy can achieve good results.^[^ ^38^, ^39^, ^40^, ^41^ ^]^ Nevertheless, given the heterogeneity of tumors and the complexity of the IGF1R signaling pathway, future studies must investigate the underlying mechanisms of IGF1R in various tumor types and assess its potential clinical application as an anti‐tumor target.

Recently, it has been found that the insulin‐like growth factor (IGF) signaling pathway plays a role in TNBC.^[^ ^42^ ^]^ IGF is highly expressed in TNBC primary tumors and it plays an important role in regulating TNBC cell growth, survival, migration and invasion.^[^ ^43^, ^44^ ^]^ IGF consists of two ligands, IGF‐I and IGF‐II, and IGF1R has high affinity for both ligands.^[^ ^45^ ^]^ The IGF1R pathway is highly active in the triple‐negative/basal‐like subtype of breast cancer.^[^ ^43^ ^]^ In addition, the interaction of IGF1R with other signaling pathways is also affecting the biological properties of TNBC. For example, the interaction of IGF1R with tumor suppressors such as p53 and PI3K may play an important role in the mechanism of drug resistance in TNBC,^[^ ^46^ ^]^ Meanwhile, IGF1R expression is strongly associated with immune cell interactions within the tumor microenvironment. Research has indicated that IGF1R activation can suppress T cell function, allowing tumor cells to evade immune surveillance and thereby enhancing tumor growth and metastasis.^[^ ^47^, ^48^ ^]^ Hence, inhibiting the IGF1R signaling pathway could boost immune cell activity and enhance the effectiveness of immunotherapy. We propose that IGF1R might act as a novel therapeutic target, potentially improving the treatment outcomes for TNBC patients when combined with immunotherapy.

The transcriptional regulation of IGF1R involves multiple layers of control. Transcription factors such as NF‐κB, SP1, and AP‐1 bind to the IGF1R promoter, modulating its expression in response to cellular signals.^[^ ^49^, ^50^, ^51^ ^]^ Epigenetic changes, such as DNA methylation and histone acetylation, also influence IGF1R expression, with hypermethylation generally suppressing it.^[^ ^52^, ^53^ ^]^ MicroRNAs like miR‐133, miR‐29, and miR‐452 target IGF1R mRNA to repress translation.^[^ ^29^, ^54^ ^]^ Growth factors and hormones, including IGF1, insulin, and growth hormone, regulate IGF1R transcription via PI3K/Akt and MAPK signaling pathways.^[^ ^55^, ^56^ ^]^ Post‐transcriptional mechanisms such as protein degradation and glycosylation further impact IGF1R stability and function. Our data suggest that SOX4 regulates IGF1R expression at the transcriptional level, supported by luciferase reporter assays, TCGA correlation analyses, and in vivo SOX4 siRNA experiments. However, due to the unavailability of ChIP‐grade SOX4 antibodies, direct evidence of SOX4 binding to the IGF1R promoter is currently lacking. This limitation prevents us from conclusively stating that SOX4 directly binds the IGF1R promoter. Future studies employing alternative approaches, such as CRISPR‐based promoter editing, could validate this interaction and further elucidate the regulatory mechanisms. This interaction underscores the critical connection between SOX4 and IGF1R in maintaining cellular homeostasis. Moreover, SOX4 is implicated in cancer progression, Its overexpression drives tumorigenesis by modulating genes related to cell growth, resistance to apoptosis, and epithelial‐to‐mesenchymal transition.^[^ ^57^, ^58^ ^]^ Understanding SOX4's role in IGF1R regulation offers insights into disease mechanisms, particularly in cancer.

The post‐translational regulation of IGF1R plays a critical role in modulating its function and stability. This regulation includes several post‐translational modifications (PTMs), such as phosphorylation, glycosylation, and ubiquitination, which influence the activity, cellular localization, and degradation of IGF1R.^[^ ^59^, ^60^, ^61^ ^]^ Glycosylation impacts receptor stability and membrane trafficking, while ubiquitination typically targets IGF1R for proteasomal degradation. These PTMs are dynamically regulated, particularly in cancer cells, where IGF1R dysregulation contributes to tumorigenesis, metastasis, and resistance to therapy.^[^ ^31^ ^]^ In our study, we discovered that DDR1, a receptor tyrosine kinase, regulates IGF1R post‐translational modifications. DDR1 interacts with IGF1R, influencing its glycosylation and ubiquitination patterns, which in turn modulate IGF1R's stability and signaling capacity. This finding highlights DDR1's dual role in mediating extracellular matrix signaling and controlling the post‐translational regulation of key receptors like IGF1R.^[^ ^62^, ^63^ ^]^ Furthermore, DDR1's upregulation in tumors suggests it may contribute to cancer progression by enhancing IGF1R‐mediated signaling. These findings shed light on the molecular mechanisms connecting DDR1 to IGF1R regulation and suggest potential therapeutic strategies for targeting DDR1 in cancer treatment.

While IGF1R is well‐known for its critical role in promoting tumor cell proliferation, survival, and therapy resistance, it also regulates various physiological functions in normal cells. In skeletal muscle, IGF1R signaling supports cell growth, regeneration, and metabolic balance.^[^ ^64^ ^]^ In endothelial cells, it is involved in angiogenesis and tissue repair, while in the immune system, IGF1R regulates the differentiation and activation of immune cells, influencing immune responses.^[^ ^65^, ^66^ ^]^ Additionally, IGF1R is vital for neuronal survival and plasticity, supporting neuronal development and function. These diverse roles underscore the broader physiological significance of IGF1R beyond tumor biology, highlighting its involvement in essential processes such as growth, metabolism, and tissue homeostasis. Understanding IGF1R's functions in normal cells provides deeper insights into its potential as a therapeutic target.

## 4 Conclusion

**Figure 8:**
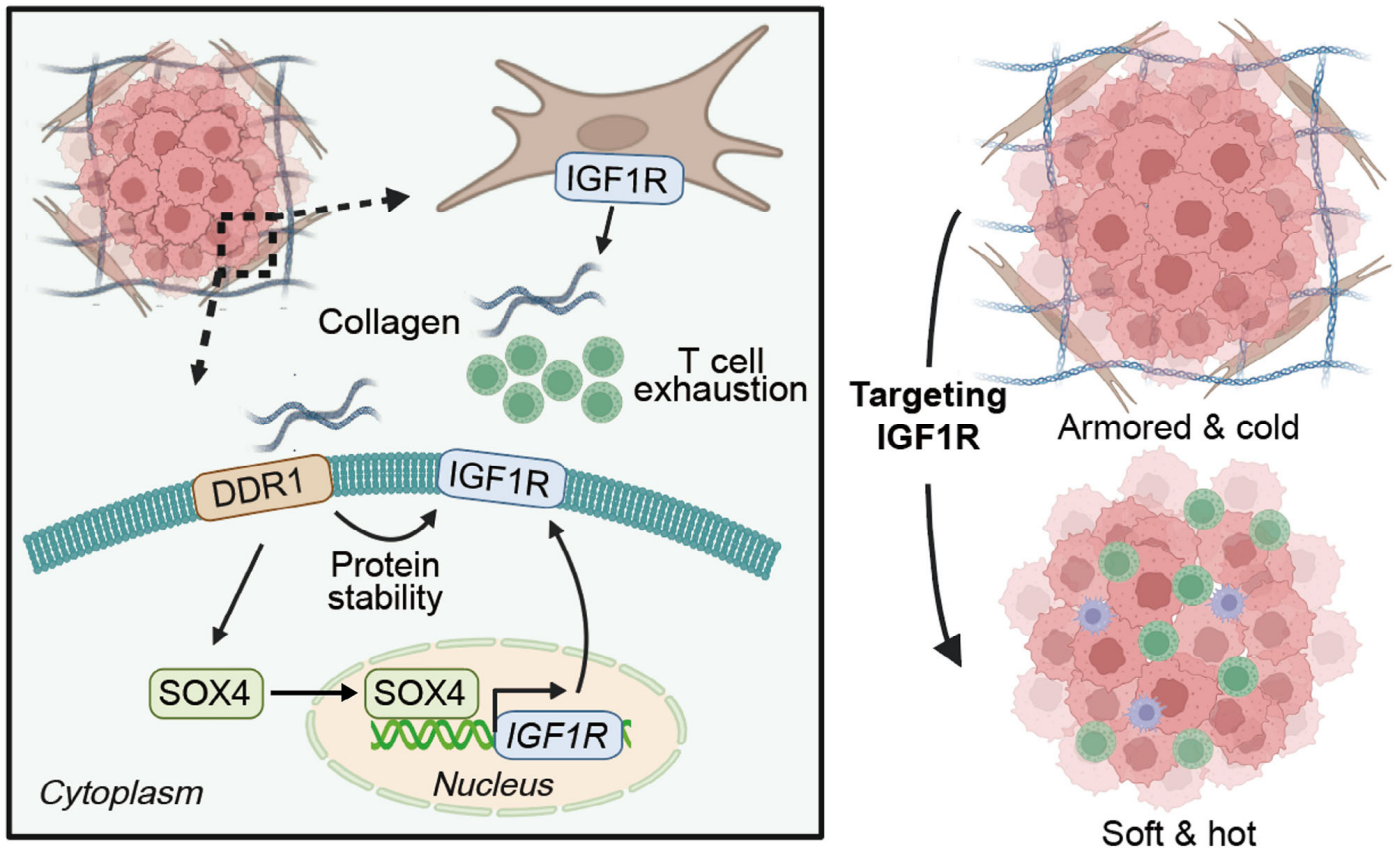
Schematic representation of the experimental content of this study. Targeted down‐regulation of IGF1R changes cold‐armored tumors into soft‐hot tumors, providing new potential immune checkpoints for TNBC treatment and laying the foundation for new combination immunotherapy regimens. In terms of molecular mechanisms, collagen regulates IGF1R expression at the transcriptional level through the transcription factor SOX4. At the same time, IGF1R interacts with the collagen receptor DDR1 to stabilize its own structure. DDR1 also promotes the nuclear translocation of SOX4, which affects IGF1R at the transcriptional level. In addition, CAFs with IGF1R expression exhibit enhanced collagen synthesis capacity. Totally, tumors with high IGF1R expression exhibit the armored & cold feature, and targeting IGF1R decreases collagen deposition and increases T cell infiltration, which turns into the soft and hot feature.

IGF1R is highly expressed in the armored & cold tumors, and collagen mediates IGF1R at the transcriptional and post‐translational levels to promote TNBC cell invasion and immune escape and to stabilize its own structure by interacting with the collagen receptor DDR1. The combination of IGF1R inhibition and PD‐1 monoclonal antibody b enhances TNBC immunotherapeutic effects. In conclusion, this study finds a new target IGF1R based on the armored & cold features and provides new molecular insights to find good immunotherapeutic options for TNBC (**Figure** **8**).

## 5 Experimental Section

### Reagents and Antibodies

The following reagents were used: Picropodophyllin (Cat. No. HY‐15494, MedChemExpress, Shanghai, China), CHX (Cat. No. HY‐12320, MedChemExpress), and 7rh (Cat. No. l9783, Sigma Aldrich, Saint Louis, USA). The following antibodies were used: anti‐IGF1R (Cat. No. 9750, Cell signaling, Boston, USA), anti‐GAPDH (Cat. No. 10494‐1‐AP, Proteintech, Wuhan, China), anti‐SOX4 (Cat. No. ab316850, Abcam, Cambridge, England), anti‐COL1A1 (Cat. No. A22090, ABclonal, Wuhan, China), anti‐DDR1 (Cat No. 5583T, CST, Boston, USA) PE anti‐CD279 (Cat.No.379210, BioLegend, California, USA), FITC anti‐GZMB (Cat. No. 372206, BioLegend, California, USA), PE anti‐CD11b (Cat.No. E‐AB‐F1081D, Elabscience, Wuhan, China). PE anti‐CD8a (Cat.No.561095, BD Bioscience, USA). APC anti‐GR1 (Cat.No. E‐AB‐F1120E, Elabscience, Wuhan, China). FITC anti‐CD3e (Cat.No. 11‐0031‐82, eBioscience, USA). The PD‐1 in vivo mAb (Cat. No. BE0273, BioXCell, Lebanon, USA).

### Clinical Samples

A paraffin‐embedded TNBC tumor microarray (TMA) (Cat. No. HBreD180Bc01) was obtained from the National Engineering Center for Biochip (Outdo Biotech, Shanghai, China). A total of 150 tumor samples and 30 para‐tumor samples were included. Ethical approval for the use of breast tumor microarray (TMA) was granted by the Clinical Research Ethics Committee in Outdo Biotech (SHYJS‐CP‐2210008).

### Immunofluorescence, mIHC, and Histochemistry Staining of Tumor Tissues

Multiplex immunohistochemistry (mIHC) was performed on paraffin‐embedded human cancer tissue samples using IHC, and HE staining for IGF1R, CD8, DDR1, and SOX4. mIHC was performed using the Think Color staining kit (FreeThinking, Nanjing, China). Mouse tumor tissues were used for immunofluorescence (IF). Primary antibodies used in mIHC and IF included: anti‐IGF1R (1:1000 dilution, Cat. No. 9750T, CST), anti‐CD8 (1:1000 dilution, Cat. No. 85336, CST), anti‐CD86 (1:1000 dilution, Cat. No. 91882, CST), anti‐CD163 (1:500 dilution, ab182422, Abcam), anti‐Ki67 (1:1000 dilution, Cat. No. ab16667, Abcam), anti‐α‐SMA (1:2000 dilution, Cat. No. 19245, CST), anti‐DDR1 (1:1000 dilution, Cat. No. 5583T, CST) and anti‐SOX4 (1:1000 dilution, Cat. No. ab316850, Abcam). Masson's trichrome staining was performed using a trichrome staining kit (Cat. No. FH115100, FreeThinking, Nanjing, China) according to the manufacturer's instructions. The results of all immunostaining were analyzed using HALO software (Indica Labs, Albuquerque, USA) and the Masson staining was quantified to determine the percentage of positively stained areas. Tumors with collagen area ≥ 10% were defined as tumors with high collagen deposition.

### Public Dataset Acquisition

Transcriptome profiles of TNBC in The Cancer Genome Atlas (TCGA) datasets were obtained from the University of California Santa Cruz Xena (https://xenabrowser.net/datapages/). A panel of public immunotherapy datasets, including the GSE173839 dataset,^[^ ^67^ ^]^ the GSE194040 dataset,^[^ ^68^ ^]^ the GSE126044 dataset,^[^ ^69^ ^]^ and the GSE176307 dataset^[^ ^70^ ^]^ comprising transcriptome data from cancer patients receiving ICB were downloaded from the Gene Expression Omnibus (GEO) database. The transcriptome data and clinical information of the IMvigor210 cohort were obtained from the official website (http://research‐pub.gene.com/IMvigor210CoreBiologies/).^[^ ^71^ ^]^ The MEDI4736 dataset was obtained from Dr. Lajos Pusztai in Yale University School of Medicine (New Haven, CT, USA).^[^ ^72^ ^]^ By combining all TNBC immunotherapy datasets (GSE173839, GSE194040, and MEDI4736), the “removeBatchEffect” function in the “limma” package.^[^ ^73^ ^]^ Gene expression changes associated with high density collagen in MBA‐MD‐231 cells were obtained from the GSE101209 dataset.^[^ ^74^ ^]^ The single‐cell RNA sequencing (scRNA‐seq) data of TNBC samples were downloaded from the GSE176078 dataset.^[^ ^75^ ^]^

### Analysis of Bulk RNA‐seq Data

The correlation of all genes with collagen score^[^ ^18^ ^]^ in the TCGA‐TNBC and the merged TNBC immunotherapy datasets were calculated. The threshold of correlation coefficient ≥ 1.5 and P < 0.05 was used. The “limma” package was utilized for the differentially expressed genes (DEGs) analysis of genes associated with responses in the merged TNBC immunotherapy dataset, and the GEO2R tool was used for the DEGs analysis of genes associated with high density collagen in MBA‐MD‐231 cells in the GSE101209 dataset. For DEGs analysis, the threshold of Fold change ≥ 1.5 and P < 0.05 was used. The transcription factors list was downloaded from the Human Transcription Factors website (http://humantfs.ccbr.utoronto.ca/).^[^ ^76^ ^]^

### Analysis of scRNA‐seq Data

The scRNA‐seq dataset of ten TNBC patients were obtained from the GSE176078 dataset.^[^ ^75^ ^]^ The high‐quality cells were reserved in which the expression of mitochondrial genes was less than 20% and with detected genes between 200 and 5,000. Subsequently, a total of 42,512 cells were used for further study. Then, the “RunHarmony” function^[^ ^77^ ^]^ was utilized to remove the biological and technical batch effects among individuals and experiments. The top 4,000 highly variable genes were used for the principal component analysis (PCA), and then the first 30 principal components (PCs) were used to reduce the dimensionality. With the 1 resolution, the 42,512 cells were unsupervised clustered into 36 groups, and then annotated into seven major cell types based on the expression distribution of conventional biomarkers.

The same steps with the same parameters were used to re‐cluster the fibroblasts acquired from the scRNA‐seq data. To explore the biological process of IGF1R+ CAF, the “FindAllMarkers” function was utilized. Genes with avg_log2FC ≥ 0.1, pct.1 ≥ 0.1 and P‐value < 0.05 were identified as IGF1R^+^ CAFs‐enriched. The R package “clusterProfiler”^[^ ^78^ ^]^ was used to investigate the enriched biological pathways based on these genes. Biological Process (BP) among Gene Ontology (GO)^[^ ^79^ ^]^ terms were identified with a strict cutoff of P‐value < 0.05. Besides, the gene set enrichment analysis (GSEA) was performed using the “GSEA” function in the “clusterProfiler” package in terms of gene signatures. The collagen signature was obtained from previous research.^[^ ^19^, ^80^ ^]^

### Cell Lines and Cell Culture

Primary CAFs were extracted from human TNBC tissues.^[^ ^19^ ^]^ Isolation of primary CD8^+^ T cells from human peripheral blood. Human cancer cell lines MDA‐MB‐231 (Cat. No. SCSP‐5043) and Hs578T (Cat. No. TCHu127) were kindly provided by Cell Bank, Chinese Academy of Sciences. Primary CAFs were cultured in a primary cell culture medium (Cat. No. CX0013, Yuchi, Shanghai, China), CD8^+^ T cells were cultured in cultured in ImmunoCult‐XF T cell expansion medium (Cat. No. 10981, STEMCELL Technologies, Vancouver, Canada). MDA‐MB‐231 cells were cultured in L‐15 (Cat. No. 11415064, Gibco, Massachusetts, USA) and Hs578T were cultured in DMEM (Cat. No.6124146, Gibco, Massachusetts, USA), and supplemented with 10% fetal bovine serum (Cat. No. Fsp500, Excell, Suzhou, China) and 1% penicillin/streptomycin (Cat. No. 15140‐122, Gibco, ThermoFisher, Massachusetts, USA). The authentication of all human cell lines was performed using short tandem repeat (STR) profiling, and it was verified that all assays were free from mycoplasma contamination.

### Cell Transfection

The siRNA (5'‐CAAUGAGUACAACUACCGCUU‐3') targeting IGF1R, the siRNA (5'‐AAGAAGGUGAAGCGCGUCUA‐3') targeting SOX4, and the siRNA (5'‐ UAUUUAUCUGAGGCCGUGU‐3') targeting DDR1 were obtained from KeyGEN (Nanjing, China). Cells were seeded into 24‐well, 6‐well, or 60 mm plates and cultured until they reached 80%‐90% confluence, after which they were transfected using Lipofectamine 3000 Transfection Reagent (Cat. No. L3000075, ThermoFisher, Massachusetts, USA).

### Real‐Time Fluorescence Quantitative PCR (RT‐qPCR)

After transfection, the cells were harvested, and total RNA was extracted with the kit (Cat. No. DP419, TIANGEN, Beijing, China). Using the SYBR Green method, the PCR assay was conducted with the following conditions: an initial denaturation at 95°C for 10 min, then 95°C for 15 s, 60°C for 1 min, 95°C for 25 s, and 60°C for 1 min, across 40 cycles. The detection primers were as follows: IGF1R, 5′‐TCGACATCCGCAACGACTATC‐3′ (F), and 5′‐CCAGGGCGTAGTTGTAGAAGAG‐3′ (R); GAPDH, 5'‐CAAATTCCATGGCACCGTCAA‐3' (F) and 5'‐AGCATCGCCCCACTTGATTT‐3' (R).

### Western Blot Analysis

Cells were lysed on ice with a laboratory‐configured 6x loading buffer. After obtaining the lysate, it was denatured at high temperatures and plated on SDS‐PAGE gels of different concentrations. After electrophoresis, the proteins were transmembraneized at 300mA. The protein bands were then incubated with antibodies and detected using enhanced chemiluminescence (ECL). The primary antibodies used as follows: anti‐IGF1R (1:1000 dilution, Cat. No. 9750, Cell signaling, Boston, USA), anti‐GAPDH (1:1000 dilution, Cat. No. 10494‐1‐AP, Proteintech, Wuhan, China), anti‐SOX4 (1:1000 dilution, Cat. No. ab316850, Abcam, Cambridge, England), anti‐COL1A1 (1:1000 dilution, Cat. No. A22090, ABclonal, Wuhan, China), and anti‐DDR1 (1:1000 dilution, Cat. No. 5583T, CST).

### Cell Proliferation Assay

The proliferative ability of cells was evaluated using the Cell Counting Kit‐8 (Cat. No. C0005, TargetMol, Shanghai, China). Cells were seeded at the appropriate density into 96‐well plates, either with or without a collagen coating on the bottom. After allowing cells to adhere, CCK‐8 reagent was added at 24 and 48 h post‐treatment. Specifically, 10 µL of CCK‐8 solution was added to each well, and the plates were incubated at constant temperature incubator for incubating cells for 1 h. Absorbance at 450 nm was then measured using a microplate reader.

For clone formation assays, 500 cells were plated per well in 6‐well plates and cultured for 10 days. After removing the culture medium, the cells were washed and fixed and stained. Finally, random fields were photographed, and colonies were counted under a microscope.

### Boyden Chamber Assay

In the small chamber assay, cells were resuspended with serum‐free medium and flattened into the upper chamber of the Transwell (Order No. 725321, NEST, Wuxi, China). The lower chamber was added with medium containing high concentration of FBS. Continue incubation in the usual culture environment for an appropriate period of time, stain the cells for fixation, and wipe off the cells in the upper chamber. If the upper chamber is coated with Matrigel (Order No. 356234, Corning, New York, USA), this step allows the determination of the invasive capacity of the cells. Cells in random areas were photographed under a microscope and their number quantified using ImageJ software.

### Wound‐Healing Assay

Seed the cells into a 6‐well plate and wait until the cells are almost full grown for the experiment. Use the yellow pipette tip to make a straight line across the cells. After scratching, wash gently with PBS and replace with fresh medium. An image of the wound area was then taken and continued to be incubated for a specific time before being taken again. The closure of the wound was quantified by measuring the remaining gap width using ImageJ software, and the relative migration rate was calculated.

### Immunofluorescence Staining of Cells

MDA‐MB‐231 and Hs578T cells were seeded into 24‐well plates for immunofluorescence analysis. After washing three times with PBS, the cells were fixed with 4% paraformaldehyde for 20 min, followed by permeabilization and blocking using a blocking buffer (Cat. No. SL1336, Coolaber, Beijing, China) at room temperature for 1 h. To assess SOX4 nuclear localization, the cells were incubated overnight at 4°C with an anti‐SOX4 antibody (1:300 dilution, Cat. No. ab316850, Abcam, Cambridge, UK) and subsequently exposed to Alexa Fluor Plus 488‐conjugated secondary antibody (1:200 dilution, Cat. No. A32723, Thermo Fisher Scientific, USA) for 1 h at room temperature. For co‐localization studies, the cells were incubated with primary antibodies targeting DDR1 (1:300 dilution, Cat. No. AF5365, Affinity, USA) and IGF1R (1:300 dilution, Cat. No. 9750, Cell Signaling Technology, Boston, USA) overnight at 4°C. This was followed by incubation with Alexa Fluor Plus 488 (1:200 dilution, Cat. No. A32723, Thermo Fisher Scientific, USA) and Alexa Fluor Plus 568 (1:200 dilution, Cat. No. A‐11004, Thermo Fisher Scientific, USA) secondary antibodies for 1 h at room temperature.

### Dual‐Glo Fluorokinase Assay

First, the SOX4 overexpression plasmid pcDNA3.1‐OE, the reporter gene IGF1R plasmid pGL3‐WT and the reporter gene point mutation plasmid pGL3‐MUT should be constructed in advance, then inoculate the appropriate number of cells into the cell culture plate, and then divide into four experimental groups to transfect the corresponding plasmids for 48 h when the cells grow to the appropriate number, then add 200 µl of lysis solution into each well of the cell culture plate and shake in a shaker for 30 min. Add 200 µl of lysate to each well of the cell culture plate, shake the plate on a shaker for 30 min, add the supernatant of each well to a 96‐well plate, and then add 50 µl of pre‐mixed Dual‐Glo Luciferase Reagent, mix well, and detect the firefly fluorescence. At the end of the assay, 50 µl of pre‐mixed Dual‐Glo Stop & Glo Reagent was added to each well, mixed well, and the sea kidney fluorescence was measured, and finally, the samples were calculated, and the data were analyzed.

### Co‐Immunoprecipitation

Cells were lysed using IP lysate (Cat. No. 87787, ThermoFisher, Massachusetts, USA). The supernatant proteins in the lysate were incubated with the antibody at 4°C overnight. After incubation of the Protein A/G‐ Agarose (Cat. No. 20421, ThermoFisher, Massachusetts, USA) for 2 h at room temperature, the beads were washed four times with lysis buffer and then lysed with 1× SDS sample buffer (Cat. No.01411, Cwbio, Taizhou, China). Samples were further subjected to Western blot analysis.

### Detection of T Cell Markers and Cytokines

Peripheral blood mononuclear cells (PBMC) from a healthy control were collected with the ethical approval from the Clinical Research Ethics Committee of The Affiliated Wuxi People's Hospital of Nanjing Medical University. Using the Dynabeads^TM^ human CD8 selection Kit (Cat. No. 11333D, Invitrogen), CD8+T cells were extracted, and they were then cultivated in ImmunoCult^TM^‐XF T cell expansion medium (Cat. No. 10981, STEMCELL Technologies). After activating T cells with ImmunoCult human CD3/CD28 T cell activator (Cat. No. 10971, STEMCELL Technologies), T cells were co‐cultured with tumor cells pre‐treated with collagen or gene inhibition at a 10:1 effector‐to‐target ratio at 37 °C for 24 h. The levels of T cell exhaustion were determined based on membrane PD‐1 expression using the corresponding antibody (Cat. No. 379210, BioLegend, California, USA) by flow cytometry analysis. For GZMB detection, cells were stained with FITC anti‐granzyme B (Cat. No. 372206, BioLegend, California, USA). For GZMB expression, the proportion of GZMB‐positive cells within CD8^+^ T cells was quantified as the percentage of GZMB⁺CD8⁺ T cells. For PD‐1 expression, the expression level was measured as relative mean fluorescence intensity (MFI) on CD8^+^ T cells. The detection of cytokines can also be used to assess the exhaustion capacity of T cells. Supernatants were collected and analyzed using IFN‐γ ELISA kit (Cat. No. E‐EL‐H0108, Elabscience, Wuhan, China) and TNF‐α ELISA kit (Cat. No. E‐EL‐H0109, Elabscience, Wuhan, China) according to the manufacturer's instructions. Absorbance was measured at 450 nm using a microplate reader.

### Animal Models

Female BALB/c mice were obtained from Zhejiang Viton Lihua Laboratory Animal Technology Co. To establish a mouse tumor model, ≈5 × 10⁶ 4T1 cells were injected subcutaneously into each mouse. Tumor dimensions were recorded using vernier calipers, and tumor volume was calculated using the formula V = (length × width^2^) × 0.5. Once the average tumor volume reached ≈100 mm^3^, the tumor‐bearing mice were randomly assigned to five groups: the control group, the PPP treatment group, the anti‐PD‐1 treatment group, and the group receiving combined PPP and anti‐PD‐1 therapy, the si‐DDR1 treatment group, and the si‐SOX4 treatment group. The control group received oral administration of phosphate buffered saline (PBS). The PPP‐treatment group received intraperitoneal injection of Picropodophyllin (20 mg kg^−1^, once a day) for 21 days. The anti‐PD‐1 treatment group was intraperitoneally injected with PD‐1 200 ug per mouse, three times a week for 21 days. The PPP‐combined anti‐PD‐1 treatment group was intraperitoneally injected with Picropodophyllin (20 mg kg^−1^, once a day) and PD‐1 200 ug per mouse, three times a week for 21 days. The si‐DDR1 treatment group received intratumoral injections of in vivo siRNA targeting DDR1 (5'‐UAUUUAUCUGAGGCCGUGU‐3', 50 µg per mouse, GeneAdv, Suzhou, GeneAdv, Suzhou,China) every three days for 12 days. The si‐SOX4 treatment group received intratumoral injections of in vivo siRNA targeting SOX4 (5'‐AAGAAGGUGAAGCGCGUCUA‐3', 50 µg per mouse, GeneAdv, Suzhou, China) every three days for 12 days. When the animal model was constructed, the mice were euthanized with carbon dioxide. Tumors were then removed from comatose animals, photographed, and weighed, and processed for further analysis. All animal studies were authorized by the Laboratory Animal Ethics Committee of Nanjing Medical University.

### Statistical Analysis

Statistical analysis was carried out using GraphPad Prism 8.0 software. Data are expressed as the mean ± standard deviation (SD) from a minimum of three independent experiments. The differences between groups were assessed using one‐way analysis of variance (ANOVA), with post hoc tests applied for multiple comparisons. An unpaired two‐tailed Student's t‐test was used for comparing two groups. A p‐value of less than 0.05 was considered statistically significant. Correlation analyses were performed using Pearson correlation coefficient.

### Ethics Approval and Consent to Participate

Ethical approval for the use of breast tumor microarray (TMA) was granted by the Clinical Research Ethics Committee in Outdo Biotech (SHYJS‐CP‐2210008). All animal experiments were approved by the Laboratory Animal Ethics Committee at Nanjing Medical University.

## Conflict of Interest

The authors declare no conflict of interest.

## Author Contributions

Y.Z., J.H., and Y.Z. conceived the study and participated in the study design and manuscript writing. M.W., J.M., and Y.C. were responsible for preparing the figures and drafting the manuscript. M.W., J.M., J.Z., N.X., and Y.J. collected and organized the experimental data. M.W., J.M., and Y.C. contributed equally to this work. All authors have read and agreed to the published version of the manuscript.

## Supporting information



Supporting Information

## Data Availability

The data that support the findings of this study are available from the corresponding author upon reasonable request.
