# Supplementary material for: Targeting IGF1R Overcomes Armored and Cold Tumor Microenvironment and Boosts Immune Checkpoint Blockade in Triple‐Negative Breast Cancer
Source: Adv Sci (Weinh). 2025 Jul 18;12(39):e01341. doi: 10.1002/advs.202501341 (PMC12533147; doi:10.1002/advs.202501341)
Supplement: Supplementary file 1 — Supporting Information [file ADVS-12-e01341-s001.docx]

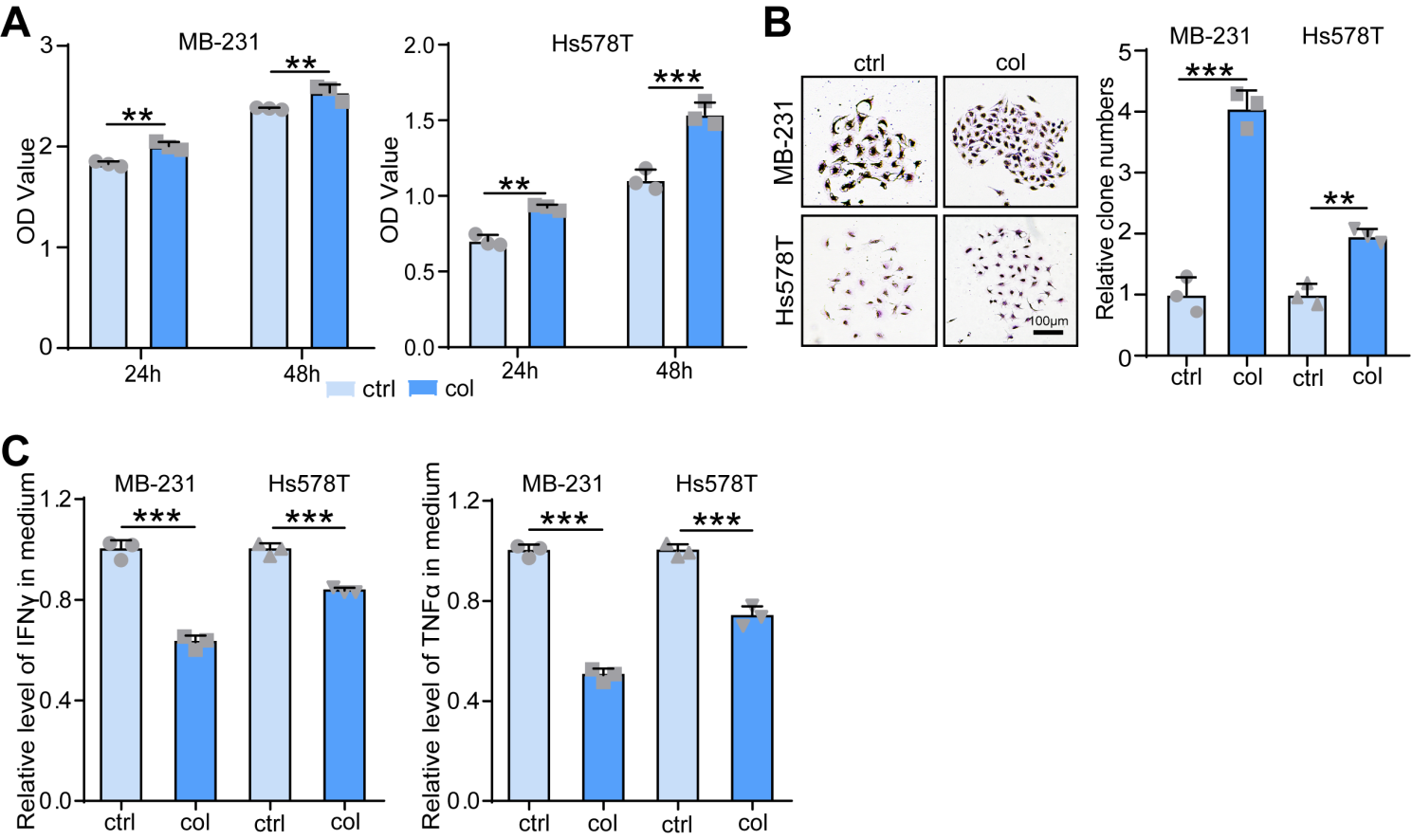


**Figure S1.** **Effects of collagen treatment on cell proliferation and T cell cytokine production. Related to Figure 1.** (A-B) The proliferation of MDA-MB-231 and Hs578T cells in ctrl and collagen-treated groups were assessed by CCK8 and colony formation assays. Data were presented as mean ± SD. Significance was calculated with Student’s t-test. All experiments were performed three times. **P < 0.01, ***P < 0.001. (C) ELISA assays for IFN-γ and TNF-α were used to assess T cell activation in the ctrl and collagen-treated groups. Data were presented as mean ± SD. Significance was calculated with Student’s t-test. All experiments were performed three times. ***P < 0.001.


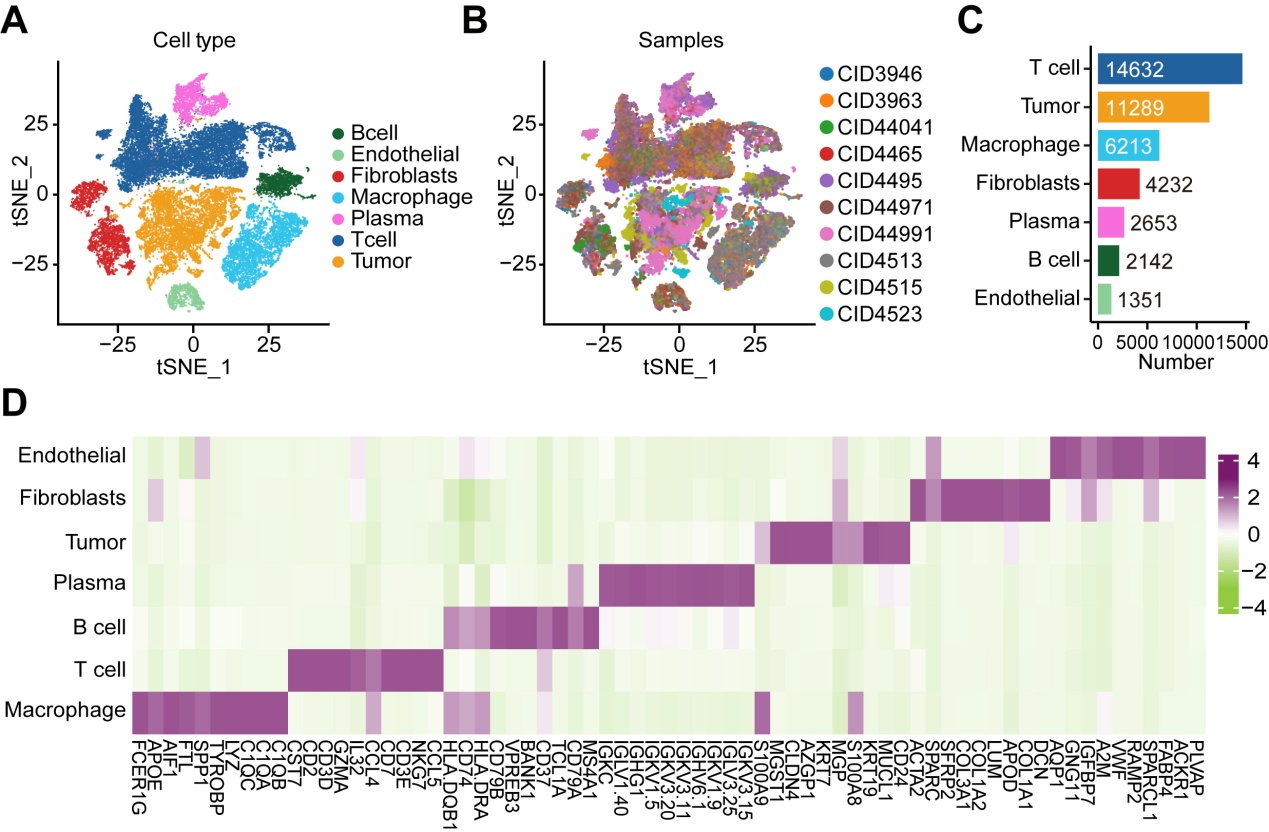


**Figure S2. Unsupervised clustering and cell type annotation of scRNA-seq data from 10 TNBC patients. Related to Figure 2.** (A) t-SNE visualization of all single cells passed quality controls, colored by 10 TNBC samples. (B) The unsupervised clustering of all single cells in various samples. (C) Bar plot showing the number of cells for each cell type. (D) Heatmap for gene expression levels of top ten cell-type-specific genes.


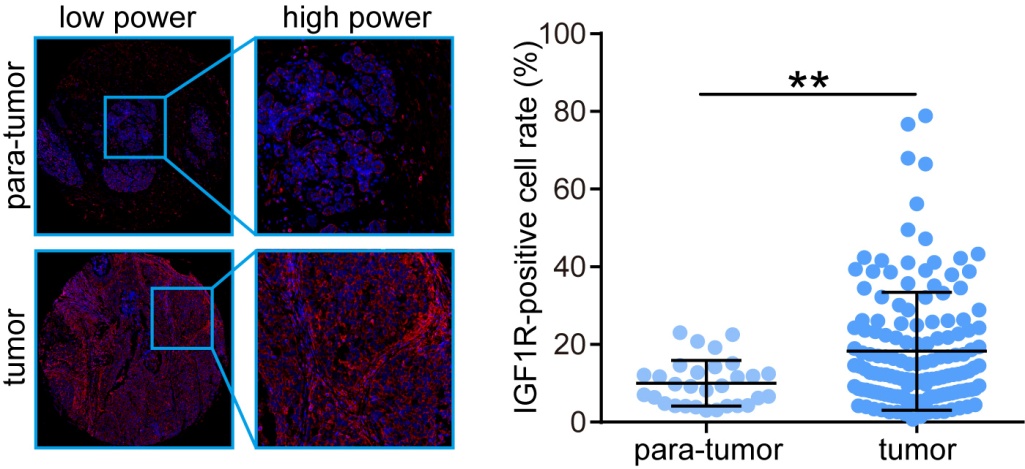


**Figure S3. Representative images uncovering IGF1R expression in para-tumor and tumor tissues in the in-house TNBC cohort and quantitative analysis. Related to Figure 2.** Significance was calculated with Mann-Whitney t-test. **P < 0.01.


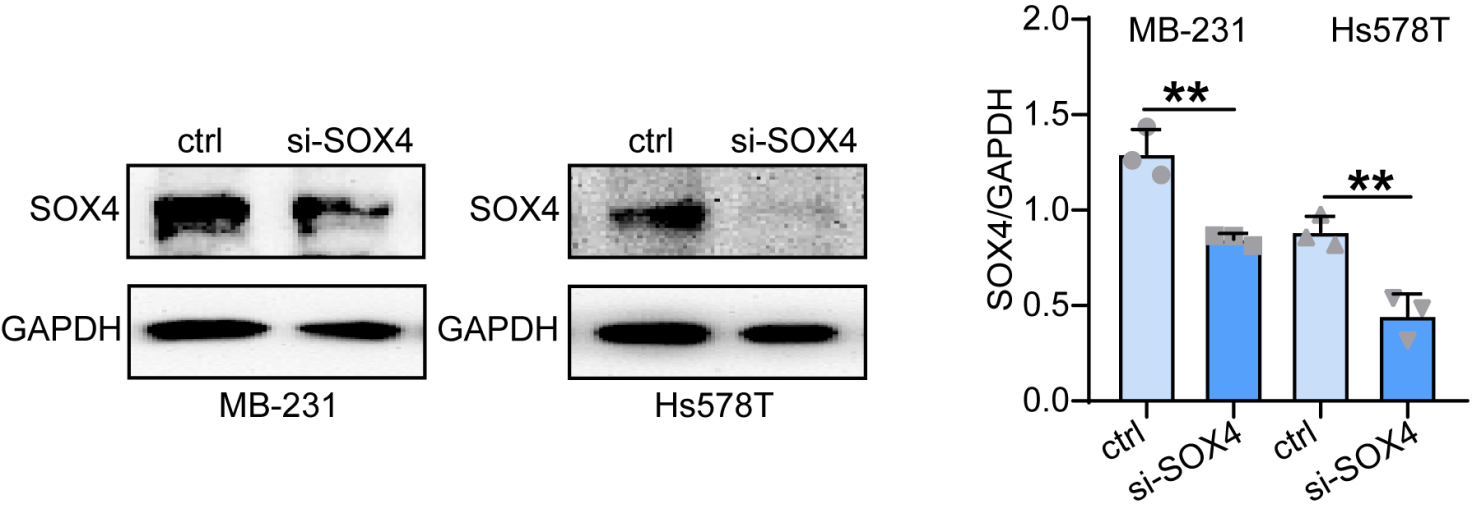


**Figure S4.** **The validation of knockdown efficiency of siRNA targeting SOX4. Related to Figure 3.** The knockdown efficiency of SOX4 in MDA-MB-231 and Hs578T cells were assessed by Western blot. The experiment was performed three times. Data was presented as mean ± SD. Significance was calculated with Student’s t-test. **P < 0.01.


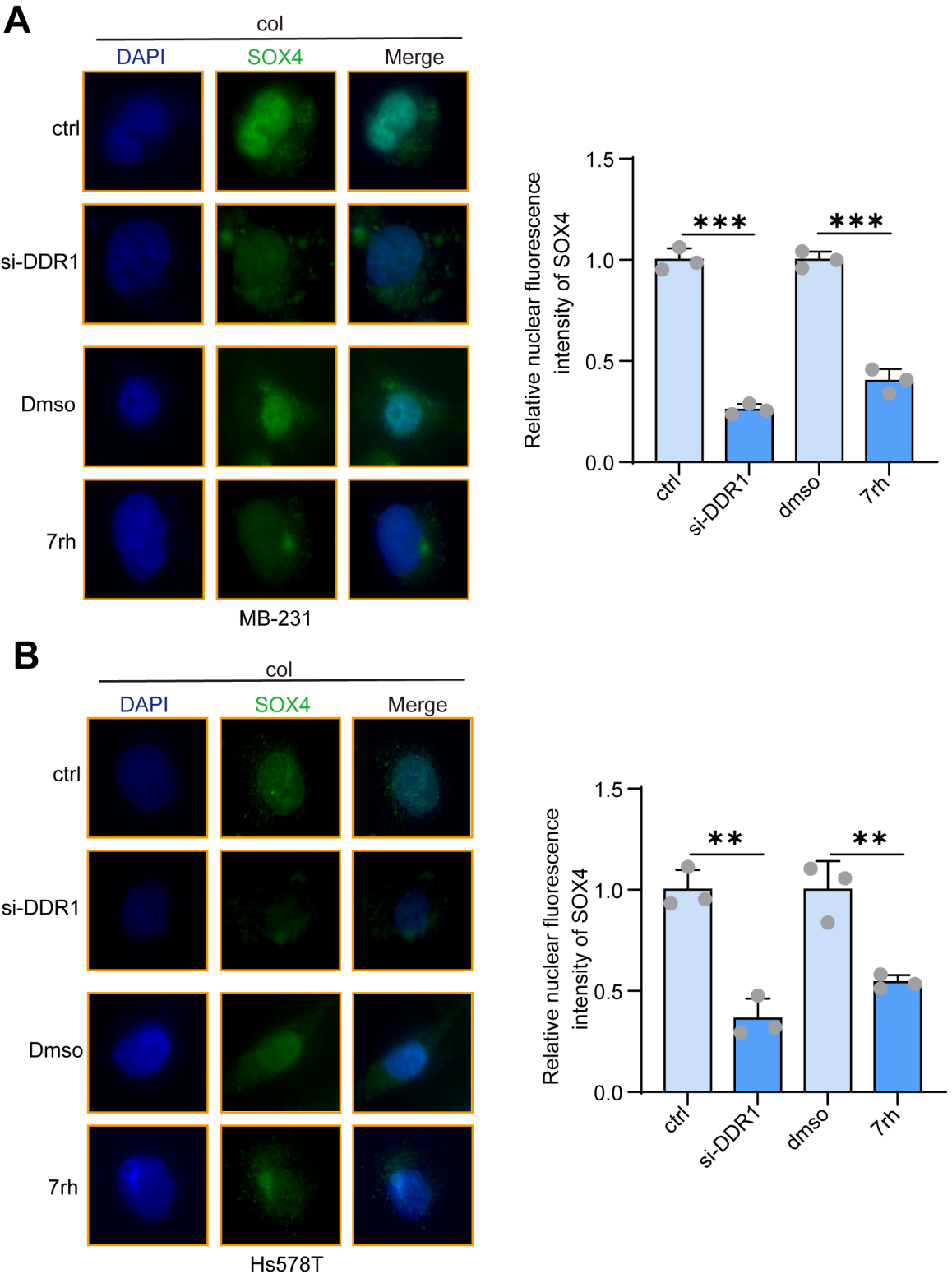


**Figure S5. Inhibition of DDR1 expression under collagen-mediated conditions affects SOX4 localization. Related to Figure 3.** (A) The location of SOX4 in si-ctrl, si-DDR1, dmso, and 7rh-treated MDA-MB-231 cells was assessed by immunofluorescence assay. Data was presented as mean±SD. Significance was calculated with the Student’s t-test test. ***P < 0.001. (B) The location of SOX4 in si-ctrl, si-DDR1, dmso, and 7rh-treated Hs578T cells was assessed by immunofluorescence assay. Data was presented as mean ± SD. Significance was calculated with the Student’s t-test test. **P < 0.01.

**
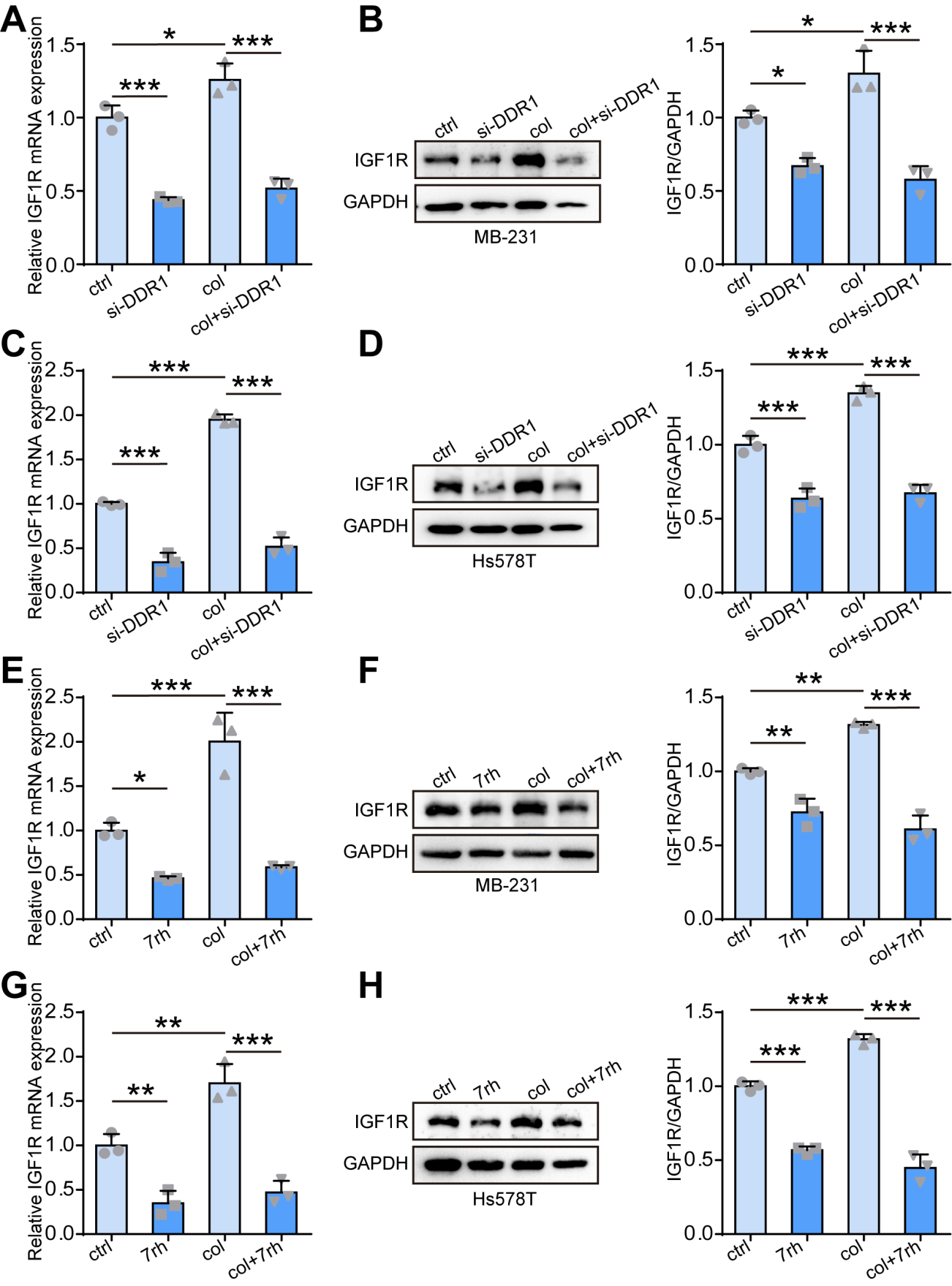
**

**Figure S6. Regulation of IGF1R protein and mRNA expression by DDR1 knockdown and 7rh treatment in collagen-treated cells. Related to Figure 4.** (A-D) Protein and mRNA expression of IGF1R in ctrl, si-DDR1, collagen-treated group and knockdown of DDR1 after collagen treatment groups assessed by western blot and qRT-PCR. Data were presented as mean ± SD. Significance was calculated with one way-ANOVA. All experiments were performed three times. *P < 0.05, ***P < 0.001. (E-H) Protein and mRNA expression of IGF1R in ctrl, 7rh-treated, collagen-treated group and 7rh-treated after collagen treatment groups assessed by western blot and qRT-PCR. Data were presented as mean ± SD. Significance was calculated with one way-ANOVA. All experiments were performed three times. *P < 0.05, **P < 0.01, ***P < 0.001.


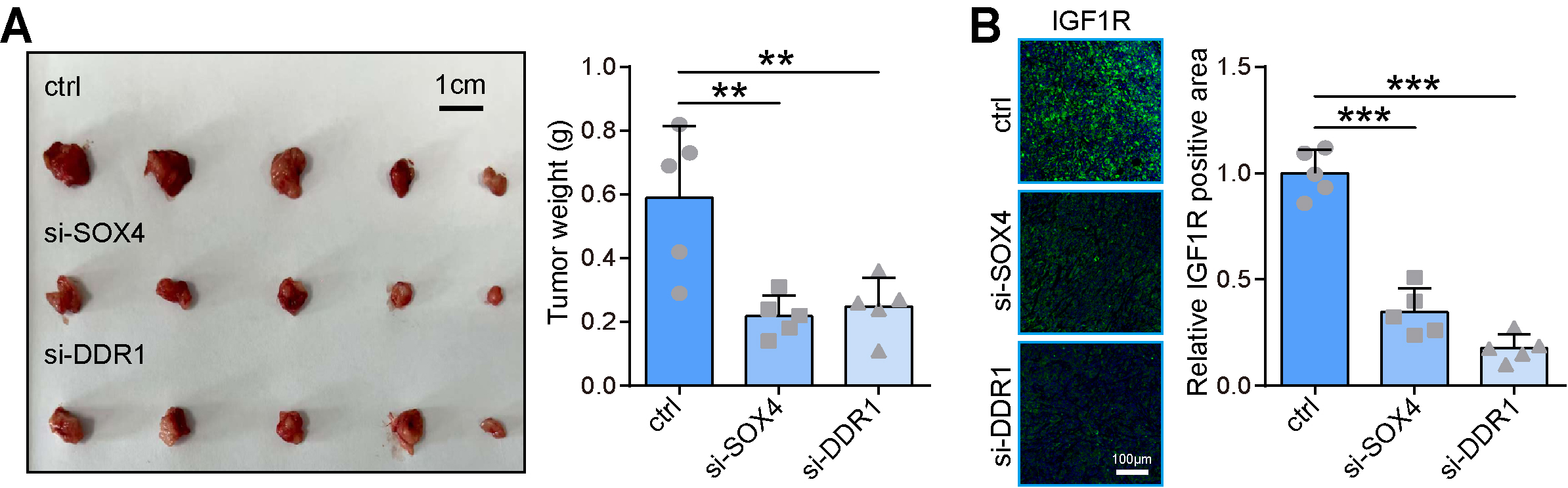


**Figure S7. The regulation of SOX4 and DDR1 on IGF1R expression in vivo.** (A) Representative images showing the tumors harvested from mice bearing 4T1 cells treated with in vivo si-SOX4 and si-DDR1, and weight of the harvested tumors. Data were presented as mean ± SD. Significance was calculated with one-way ANOVA. **P < 0.01. (B) Representative images showing IGF1R levels in tumor tissues from different groups of mice and quantitative analysis. Data were presented as mean ± SD. Significance was calculated with one-way ANOVA. ***P < 0.001.


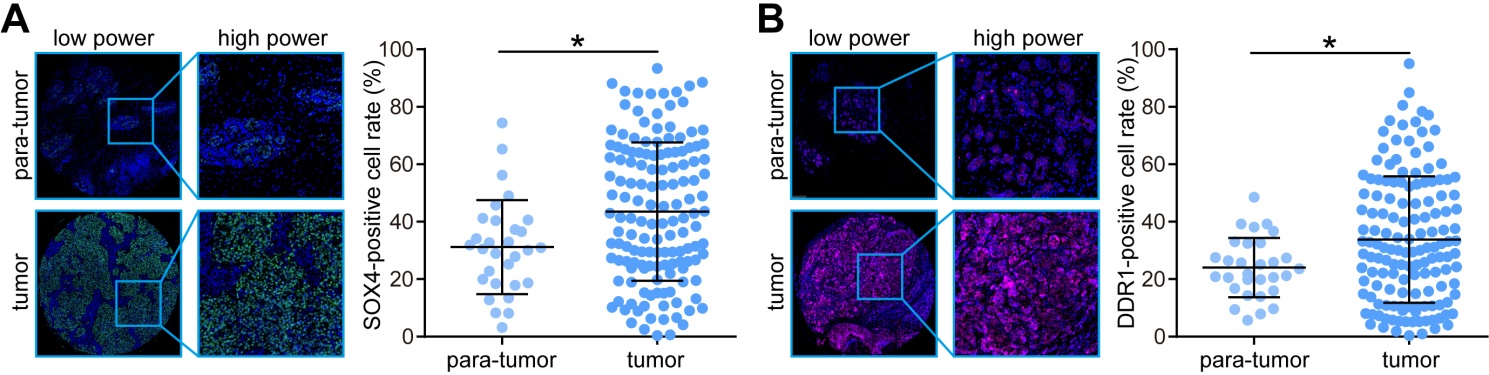


**Figure S8. Expression of SOX4 and DDR1 in para-tumor and tumor tissues. Related to Figure 3 and Figure 4.** (A) Representative images uncovering SOX4 expression in para-tumor and tumor tissues in the in-house TNBC cohort and quantitative analysis. Significance was calculated with Mann-Whitney t-test. *P < 0.05. (B) Representative images uncovering DDR1 expression in para-tumor and tumor tissues in the in-house TNBC cohort and quantitative analysis. Significance was calculated with Mann-Whitney t-test. *P < 0.05.


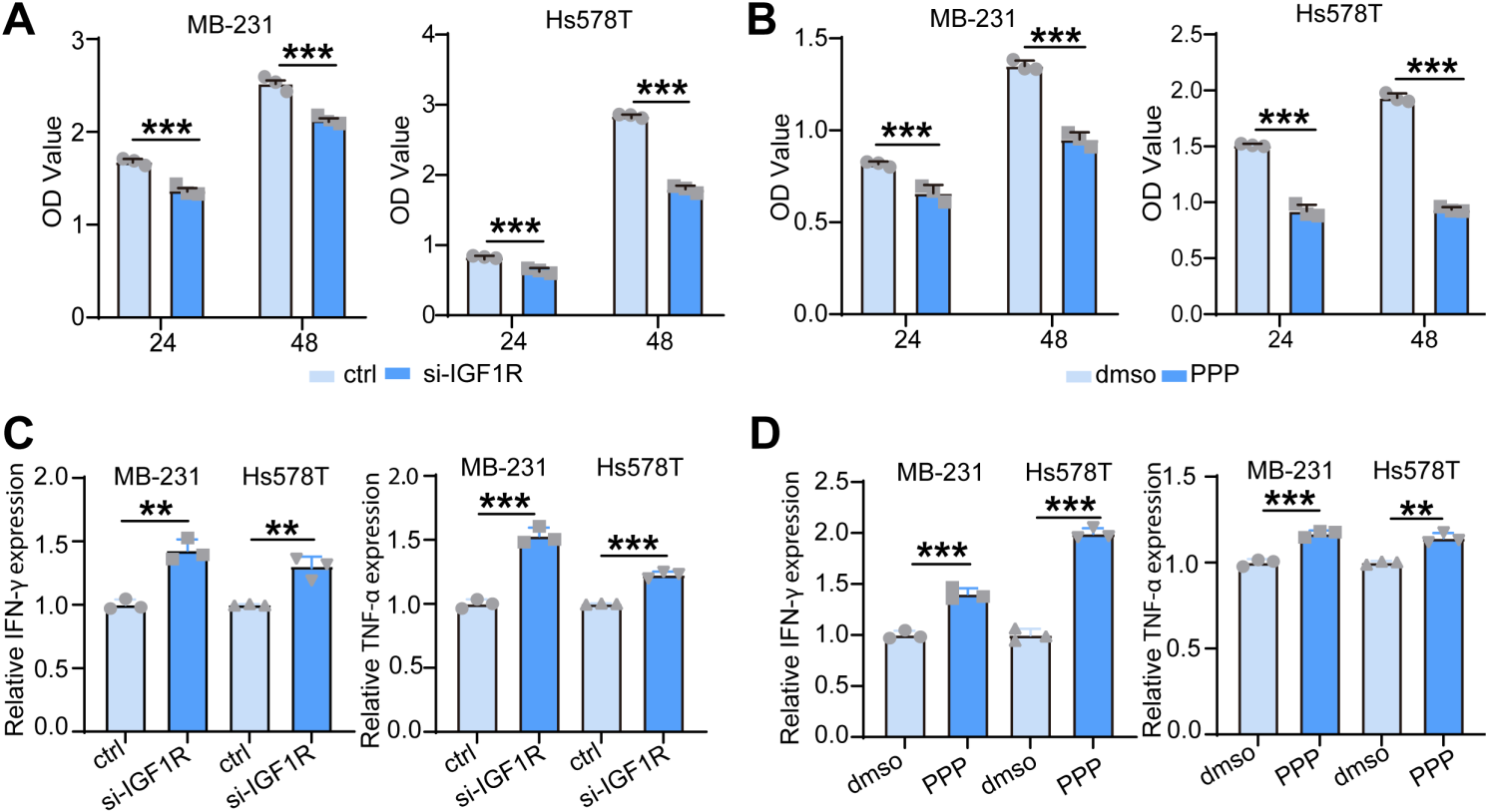


**Figure S9. Impacts of IGF1R knockdown and PPP treatment on cell proliferation and T cell cytokine production in MDA-MB-231 and Hs578T cells. Related to Figure 5.** (A-B) The proliferation of MDA-MB-231 and Hs578T cells in ctrl, si-IGF1R, dmso, and PPP-treated groups were assessed by CCK8 assay. Data were presented as mean ± SD. Significance was calculated with Student’s t-test. All experiments were performed three times. ***P < 0.001. (C-D) ELISA assays for IFN-γ and TNF-α levels were used to assess T cell activation in ctrl, si-IGF1R, dmso, and PPP-treated groups in MDA-MB-231 and Hs578T cells. Data were presented as mean ± SD. Significance was calculated with Student’s t-test. All experiments were performed three times. **P < 0.01, ***P < 0.001.

**
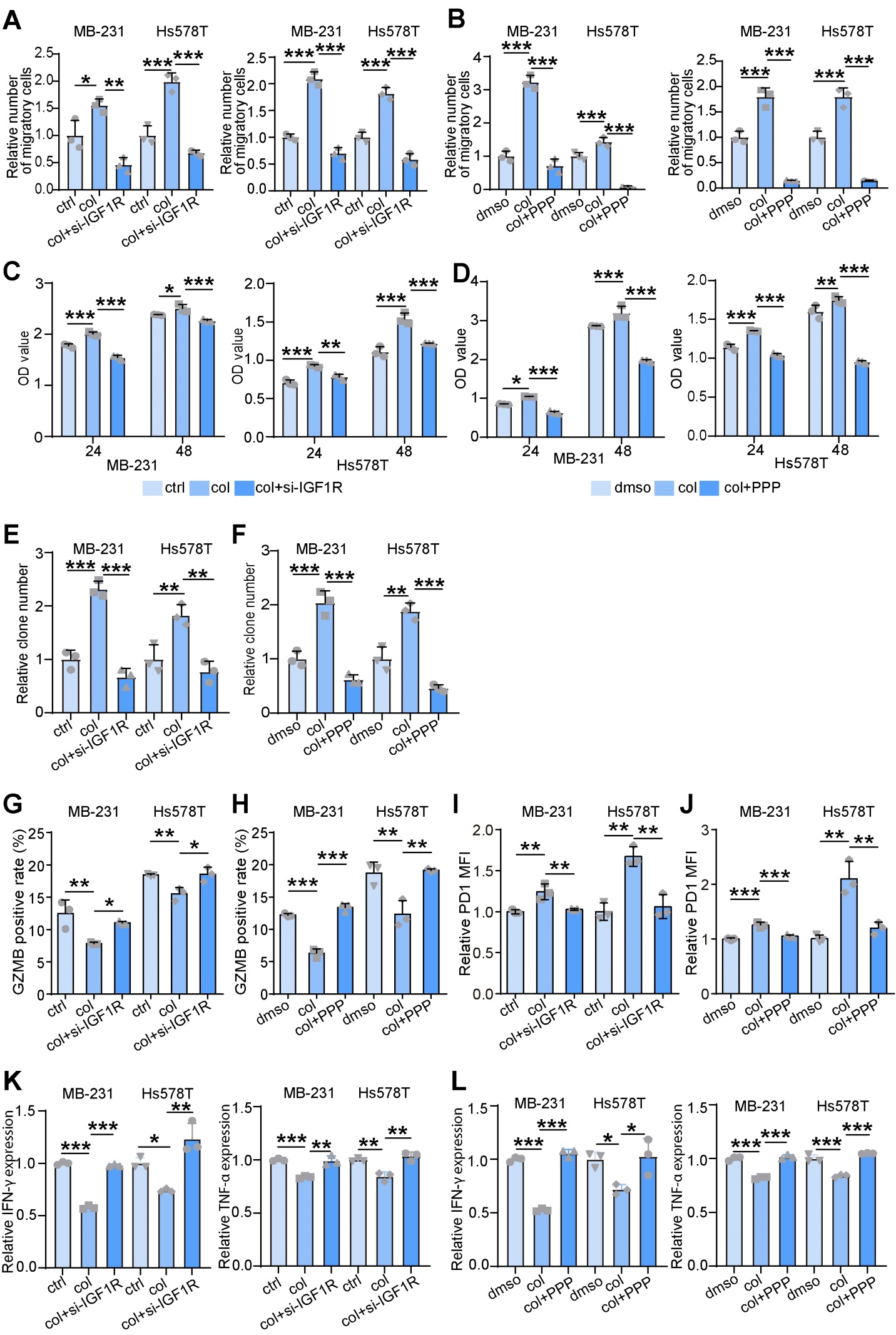
**

**Figure S10.** **IGF1R is a collagen-mediated regulator of tumor progression and T cell exhaustion.** (A) The migration and invasion of MDA-MB-231 and Hs578T cells in control, collagen, and knockdown of IGF1R (si-IGF1R) after collagen treatment groups were assessed by Boyden chamber assays. Data were presented as mean ± SD. Significance was calculated with one way-ANOVA. All experiments were performed three times. *P < 0.05, **P < 0.01, ***P < 0.001. (B) The migration and invasion of MDA-MB-231 and Hs578T cells in dmso, collagen + dmso, PPP and collagen co-treated groups were assessed by Boyden chamber assays. Data were presented as mean ± SD. Significance was calculated with one way-ANOVA. All experiments were performed three times. ***P < 0.001. (C) The proliferation of MDA-MB-231 and Hs578T cells in ctrl, collagen-treated, and knockdown of IGF1R after collagen treatment groups were assessed by CCK8 assay. Data were presented as mean ± SD. Significance was calculated with one way-ANOVA. All experiments were performed three times. *P < 0.05, **P < 0.01, ***P < 0.001. (D) The proliferation of MDA-MB-231 and Hs578T cells in dmso, collagen + dmso, PPP and collagen co-treated groups were assessed by CCK8 and colony formation assays. Data were presented as mean ± SD. Significance was calculated with one way-ANOVA. All experiments were performed three times. *P < 0.05, **P < 0.01, ***P < 0.001. (E) The proliferation of MDA-MB-231 and Hs578T cells in control, collagen, and knockdown of IGF1R (si-IGF1R) after collagen treatment groups were assessed by colony formation assays. Data were presented as mean ± SD. Significance was calculated with one way-ANOVA. All experiments were performed three times. **P < 0.01, ***P < 0.001. (F) The proliferation of MDA-MB-231 and Hs578T cells in dmso, collagen + dmso, PPP and collagen co-treated groups were assessed by colony formation assays. Data were presented as mean ± SD. Significance was calculated with one way-ANOVA. All experiments were performed three times. **P < 0.01, ***P < 0.001. (G, I) Flow cytometry was performed to detect GZMB and PD1 levels in the control, collagen, and knockdown of IGF1R (si-IGF1R) after collagen treatment groups. Data were presented as mean ± SD. Significance was calculated with one way-ANOVA. All experiments were performed three times. *P < 0.05, **P < 0.01. (H, J) Flow cytometry was performed to detect GZMB and PD1 in dmso, collagen + dmso, PPP and collagen co-treated groups. Data were presented as mean ± SD. Significance was calculated with one way-ANOVA. All experiments were performed three times. **P < 0.01, ***P < 0.001. (K) ELISA assays for IFN-γ and TNF-α levels were used to assess T cell activation in ctrl, collagen-treated, and knockdown of IGF1R after collagen treatment groups in MDA-MB-231 and Hs578T cells. Data were presented as mean ± SD. Significance was calculated with one way-ANOVA. All experiments were performed three times. *P < 0.05, **P < 0.01, ***P < 0.001. (L) ELISA assay for IFN-γ and TNF-α levels were used to assess T cell activation in dmso, collagen + dmso, PPP and collagen co-treated groups in MDA-MB-231 and Hs578T cells. Data were presented as mean ± SD. Significance was calculated with one way-ANOVA. All experiments were performed three times. *P < 0.05, ***P < 0.001.


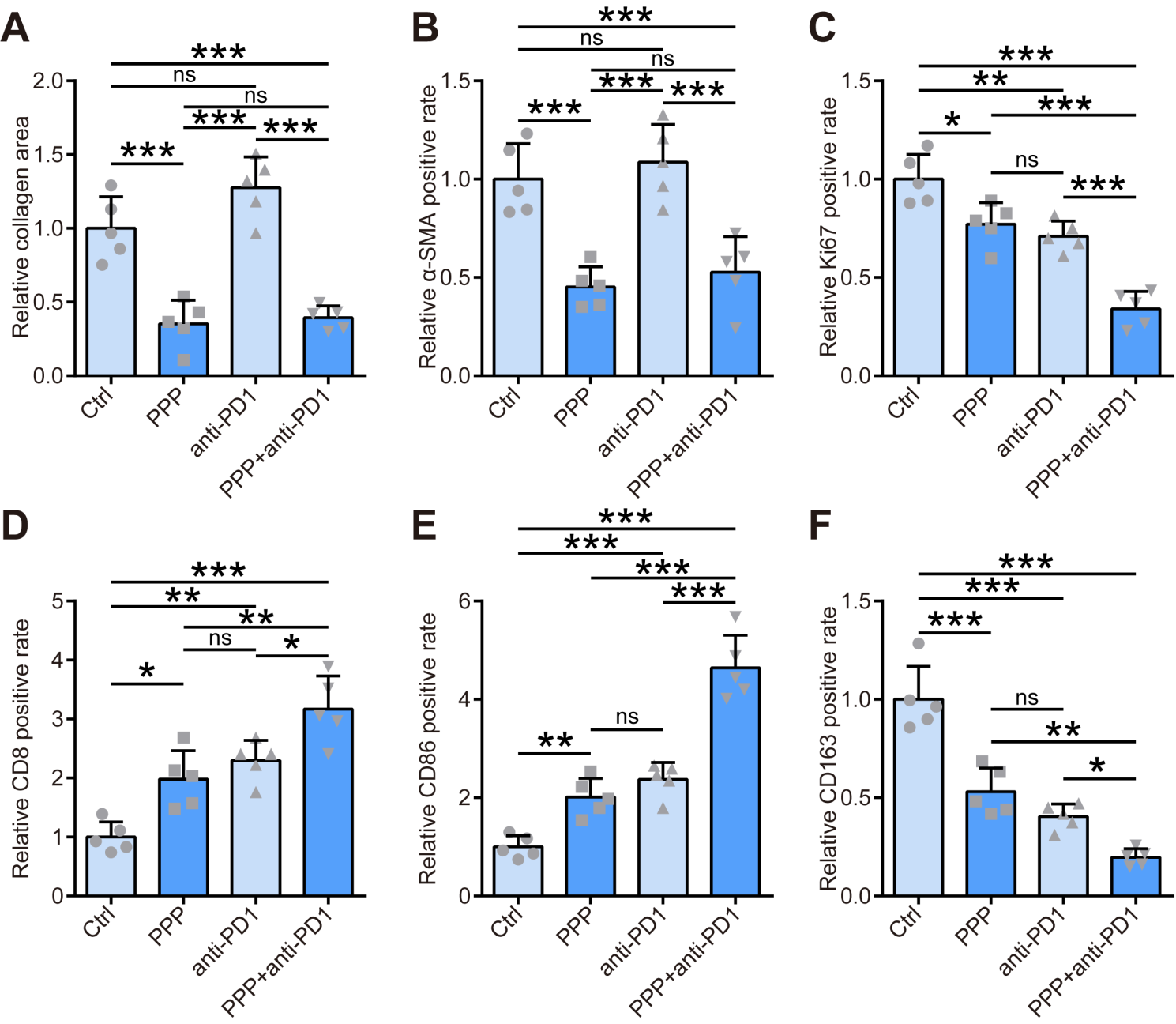


**Figure S11. Effects of PPP and anti-PD1 treatment on collagen area, α-SMA, Ki67, CD8, CD68, and CD163 expression in treated groups. Related to Figure 7.** (A) Collagen area. (B) α-SMA. (C) Ki67. (D) CD8. (E) CD86. (F) CD163. Data were presented as mean ± SD. Significance was calculated with one way-ANOVA. All experiments were performed in five samples. *P < 0.05, **P < 0.01, ***P < 0.001, ns, no significance.


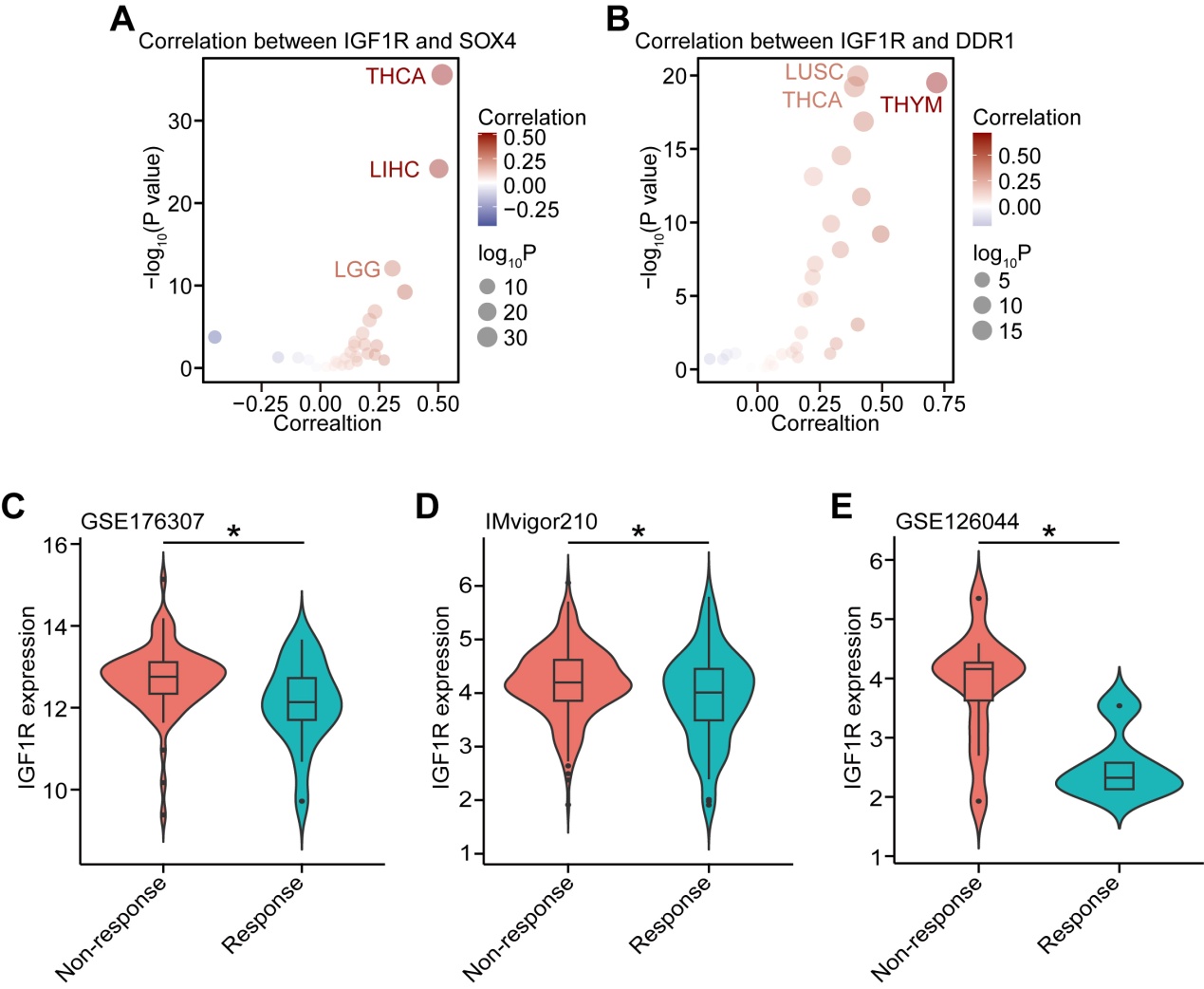


**Figure S12. Pan-cancer analysis of IGF1R using the TCGA and immunotherapy datasets.** (A, B) Correlations between IGF1R and SOX4 as well DDR1 in different cancer types and their significance, correlations are indicated by color shades (red is a positive correlation, blue is a negative correlation). The p-value is displayed by dot size. The p-value is displayed by dot size. (C-E) Differences in IGF1R expression in the GSE176307 dataset, the IMvigor210 dataset, and the GSE126044 dataset between the non-responders and responders. Significance was calculated with Student’s t-test. *P < 0.05.
